# Investigating the effects of continuous theta-burst stimulation over the posterior parietal cortex on holistic processing of composite faces: evidence from cognitive modeling

**DOI:** 10.1371/journal.pone.0343776

**Published:** 2026-03-23

**Authors:** Hanieh Sayadi, Hasan Qarehdaghi, Hamidreza Pouretemad, Jamal Amani Rad

**Affiliations:** 1 Institute for Cognitive and Brain Sciences, Shahid Beheshti University, Tehran, IRAN; 2 Choice Modelling Centre, University of Leeds, Leeds, United Kingdom; Hangzhou Normal University, CHINA

## Abstract

Efficient interaction with complex visual environments depends on the balance between global and local processing, with a natural tendency to prioritize global information—a phenomenon known as the global advantage. This study investigates how non-invasive brain stimulation using continuous theta-burst stimulation (cTBS) over the posterior parietal cortex (PPC) affects this balance, with a focus on holistic face processing. We conducted a within-subject, crossover, sham-controlled experiment involving 36 participants who completed Navon and composite face tasks before and after cTBS stimulation over the left and right PPC. Cognitive modeling using the Diffusion Model of Conflict (DMC) was employed to examine the cognitive mechanisms involved and compare global and local processing in experimental versus sham conditions. The results confirmed the expected global precedence effect and holistic processing in both tasks but revealed no significant impact of cTBS over the right PPC on task performance in terms of accuracy or response time. However, under incongruent conditions, cTBS over the left PPC led to slower responses to local targets, particularly when compared with the sham condition. Despite these localized effects, no significant changes were observed in composite face task performance following either left or right PPC stimulation. Overall, our findings highlight the complexity of the neural mechanisms governing global-local processing and the limited impact of single-session cTBS over the PPC in altering the cognitive mechanisms underlying these processes. Further research is needed to better understand the role of the PPC in holistic and hierarchical visual processing.

## Introduction

Most visual stimuli encountered in daily life are hierarchically structured, composed of multiple elements arranged in specific spatial configurations. Each element within such stimuli can be interpreted as either relatively global or local in nature, depending on its spatial scale and context [1,2]. For instance, in an image of a mountain range, the mountains and rivers form the global level, while smaller details like stones or trees constitute the local level. Similarly, a tree may serve as the global level, while individual leaves are representative of the local level. Adaptive visual processing requires the ability to integrate these discrete elements into coherent global forms, while simultaneously preserving the distinct characteristics of local components [3–5].

To investigate how attentional resources are allocated between global forms and local details, researchers have employed hierarchical stimuli, such as the well-known “Navon stimuli” [3,6]. These stimuli typically consist of large letters (global level) constructed from smaller letters (local level), with the relationships between the levels manipulated to create congruent or incongruent conditions. Hierarchical stimuli may also take the form of shapes [e.g., 7, 8] or objects [e.g., 9], and can be used in selective attention paradigms where participants are instructed to identify targets at one level, or in divided attention tasks where targets may appear at either level. Importantly, the global and local levels are designed to be equally identifiable and mutually independent [3,10], allowing for an unbiased measurement of global and local processing [11].

The Navon task consistently demonstrates the “global advantage effect" (GA), wherein participants respond more rapidly to global elements compared to local ones. Furthermore, in incongruent trials, global features tend to interfere with the identification of local elements—a phenomenon known as global-to-local interference (GLI)—while local features do not exert the same disruptive effect on global processing [3,10]. However, research has shown that this global processing advantage is not universally observed [4]. Variations have been documented in individuals with certain neurodevelopmental disorders [12–15], across demographic groups differing in sex, hormonal cycles [16–18], ethnicity, and cultural backgrounds [19–22]. Additionally, priming [23–27] and attentional demand [28] can modulate this effect. Moreover, the magnitude and even the direction of the global precedence effect can be influenced by manipulations in stimulus or task characteristics, such as larger visual angles [5,11,29], shorter exposure durations [30], reduced spacing between local elements [31,32], and lower densities of local elements [2,4,33,34].

The local-global dichotomy central to Navon stimuli has also been explored in the context of face processing, an area where holistic mechanisms are critical. Configural processing—recognizing the spatial relationships among facial features—is as essential to face perception as feature-based processing, which isolates individual components of the face. There is evidence to suggest that global precedence effects contribute similarly to the facilitation of both face and object recognition which means that face processing and Navon hierarchical letters have a remarkable resemblance and are driven by similar holistic mechanisms [35,36]. For example, in both Navon letter tasks and face processing, the global structure—the overall face—takes precedence over local components such as the eyes or nose [37,38]. Consequently, any external factor that disrupts configural face processing is likely to affect performance on hierarchical Navon stimuli as well. This observation has motivated contemporary research to explore factors that modulate the balance between local and global processing.

This study aims to explore the mechanisms underlying the global-local dichotomy, emphasizing their implications for face and object processing. In doing so, we address the interplay between these processes and their susceptibility to manipulation by external factors, such as non-invasive brain stimulation. In addition to traditional experimental approaches, cognitive modeling provides a powerful framework for exploring the mechanisms underlying the global-local dichotomy, emphasizing their implications for face and object processing. By mathematically modeling the cognitive and temporal dynamics underlying these processes, cognitive models—such as diffusion decision models and evidence accumulation frameworks—offer insights that go beyond surface-level behavioral outcomes [39,40]. These models quantify how perceptual and decisional processes interact, enabling a deeper understanding of how external manipulations, such as non-invasive brain stimulation, bias global and local processing orientations. Incorporating cognitive modeling not only complements behavioral findings but also bridges the gap between observed effects and their underlying cognitive mechanisms.

### Measuring holistic face processing

Building on the principles of local and global processing discussed above, it becomes clear that certain perceptual tasks—such as face recognition—demand a more holistic approach. Holistic processing refers to the tendency to perceive individual features as an integrated perceptual whole, a process particularly critical for distinguishing visually similar objects such as faces [41–45]. This form of processing sets face recognition apart as a unique form of object recognition, involving perceptual mechanisms that are rarely observed for non-face objects [41,45]. Although both feature-based processing (isolated recognition of individual facial components) and configural processing (understanding the spatial relationships between features) contribute to face recognition, holistic processing is considered the dominant mechanism [46–48]. One hallmark of holistic processing is the ability to recognize faces at the level of individual identity (e.g., recognizing “Michael”) rather than merely categorizing them at a broader level (e.g., recognizing a “human face”) [49]. This individualized processing enables the discrimination of faces that share common features, such as eyes, noses, and mouths arranged in the same general configuration.

The composite face effect is one of the most robust paradigms for measuring holistic processing [45–47,50–56]. This paradigm creates composite faces by combining the upper half of one face with the lower half of another, producing a novel identity when these halves are aligned. In a typical composite face task, participants are presented with pairs of such faces and instructed to focus only on the top half while ignoring the bottom half [52,57,58]. The illusion of a fused identity often interferes with selective attention, impairing participants’ ability to make same/different judgments about the top halves of the faces [56,58–61]. This interference, referred to as the alignment effect, diminishes when the bottom half is misaligned or horizontally offset. The disruption of the meaningful facial configuration under these conditions directly impacts holistic processing, as the perceptual integration of facial parts is weakened [47,52,53,56,57,62–67]. For example, presenting a composite face with the top half of George Clooney and the bottom half of Tony Blair slows response times and increases errors in aligned conditions compared to misaligned ones [56].

Two primary variations of the composite face task—partial and complete designs—have been used to quantify holistic processing (see [68,69]). The partial design, originally introduced by [56,57], compares performance in trials where the bottom half is always different but the top half may be either the same or different. Holistic processing is inferred from the alignment effect, which is typically observed only when the top halves are identical. In contrast, the complete design [41] allows for both the top and bottom halves of composite faces to vary independently [43,55], creating congruent and incongruent trials. Here, holistic processing is measured by the interaction between alignment and congruency. Specifically, the alignment effect is reduced in incongruent trials, where the irrelevant bottom half of the face conflicts with the target identity in the top half [45, 70, 71]. This approach offers a more nuanced measure of holistic processing compared to the partial design.

Recent research [45, 65, 70, 71, 72] favors the complete design for several reasons. First, the partial design is susceptible to response biases because the irrelevant bottom half is always different, creating an association between congruency and response type [45,65,70,71]. Second, the partial design has demonstrated weak correlations with other measures of holistic processing, such as the part-whole task [73,74]. In contrast, the factorial structure of the complete design mitigates these limitations by systematically manipulating both alignment and congruency, yielding results that are more reliable and generalizable [70–72]. More importantly, cognitive modeling provides a critical extension to these behavioral paradigms, offering a framework to dissect the cognitive mechanisms underlying holistic face processing. For example, by quantifying the interaction between congruency and alignment, such models can shed light on how perceptual integration, evidence accumulation, and decision-making strategies influence performance in composite face tasks, which are not directly observable but critically shape holistic interference. These approaches provide a structured framework to explore how experimental manipulations, like alignment offsets, affect the cognitive processes driving holistic interference. Incorporating modeling into this context not only enhances our understanding of holistic face processing but also facilitates the connection between task design, experimental manipulations, and specific cognitive mechanisms.

In this study, we adopted the complete design to measure holistic face processing. By employing this approach, we aimed to capture the interplay between congruency and alignment, thereby providing a robust index of holistic face processing and its potential disruption under specific experimental manipulations.

### Do manipulating factors lead to local and global processing bias?

Having established the foundations of holistic and hierarchical processing, an important question arises: can external factors systematically bias the adoption of local or global processing strategies? If global processing is essential for holistic perception, any factor that induces a shift toward local processing may disrupt holistic face recognition and vice versa. This hypothesis has received empirical support from studies employing priming and other experimental manipulations [23–25].

Priming, in particular, has been widely used to modulate processing strategies [23–25]. The Navon task, with its inherent dichotomy between global and local levels, has frequently served as a priming tool to induce shifts in attention. For example, Macrae and Lewis [75] demonstrated that participants exposed to Navon stimuli for 10 minutes at either the global or local level subsequently exhibited processing biases that affected face recognition. Specifically, participants who shifted from global to local processing were significantly impaired in identifying previously viewed faces, suggesting that the activation of local processing disrupts holistic perception. This effect has been replicated in composite face studies, where local priming has been shown to enhance recognition of individual facial components without improving overall accuracy [23–25].

However, the duration and robustness of priming effects remain contentious. While some studies report lasting effects of global or local priming [24], others suggest that these effects are transient, decaying rapidly after priming ceases [76,77]. For instance, Hills and Lewis [76] observed that Navon task priming effects diminished within one minute or after approximately 20 trials. This rapid decline suggests that priming, though effective in momentarily altering processing orientation, may lack the durability needed for long-term modulation. Furthermore, inconsistencies have been reported in how priming affects congruent versus incongruent trials in composite face tasks. For example, Gao et al. [24] found that global priming heightened sensitivity to congruency effects, but local priming had no significant impact on congruent or incongruent trials.

These limitations underscore the need for alternative methods that offer more robust and lasting effects on processing biases. Given these findings, researchers have explored non-invasive brain stimulation techniques, such as transcranial magnetic stimulation (TMS), to systematically bias processing strategies. Unlike priming, TMS allows for direct modulation of neural activity in targeted cortical areas, offering a more precise and potentially long-lasting method for altering processing orientations [78–81]. By disrupting or enhancing activity in specific brain regions, TMS has been shown to bias attention toward either global or local levels of information. For instance, Romei et al. [79] used rhythmic TMS over the posterior parietal cortex to modulate processing orientation in hierarchical Navon tasks, finding that beta-frequency stimulation over the right PPC enhanced local processing, while theta-frequency stimulation enhanced global processing.

Although neuroimaging studies provide correlational insights into the neural mechanisms underlying local and global processing, TMS enables causal investigations. By inducing temporary “virtual lesions,” TMS allows researchers to examine how disruptions in specific cortical regions affect processing biases [82,83]. Compared to priming, TMS offers the advantage of lasting effects and greater specificity, making it a powerful tool for understanding the neural substrates of local and global processing and their role in holistic perception. Cognitive modeling complements these causal investigations by quantifying how neural disruptions translate into changes in decision-making dynamics. For instance, modeling approaches allow researchers to examine how TMS-induced biases influence the integration of global and local information over time, providing a mechanistic understanding of processing shifts that extends beyond behavioral outcomes.

In light of these advancements, the current study employs TMS to investigate how neural stimulation can induce and sustain biases in local and global processing. By targeting the posterior parietal cortex—a region implicated in attentional allocation and hierarchical processing—this approach seeks to address the limitations of priming while providing a robust framework for manipulating cognitive orientations over time.

### Neural mechanisms underlying hemispheric differences: Current insights and research gaps

The neural mechanisms underlying global and local visual processing reveal a consistent pattern of hemispheric specialization. Over the years, a growing body of evidence has demonstrated that global processing is predominantly governed by the right hemisphere, while local processing is largely facilitated by the left hemisphere. This functional hemispheric asymmetry (FHA) has been supported by research spanning neuropsychological studies, electrophysiological investigations, and functional neuroimaging [84, 85]. Together, these findings highlight the critical role of the hemispheres in segregating and integrating visual information at different levels.

The earliest insights into FHA came from neuropsychological studies of patients with brain injuries. Individuals with lesions in the right hemisphere often exhibit impairments in global processing, while their ability to process local details remains intact [86–89]. Conversely, damage to the left hemisphere typically results in deficits in local processing without significant disruption to global perception. These observations provided foundational evidence for the lateralization of global and local processing mechanisms.

Further support for this hemispheric specialization has come from functional imaging studies in healthy participants. Using techniques such as functional magnetic resonance imaging (fMRI) and positron emission tomography (PET), researchers have consistently shown that the right posterior parietal cortex is preferentially activated during global processing tasks, whereas the left PPC is more involved in local processing [8, 85, 90–97]. These findings have been bolstered by event-related potential (ERP) studies, which indicate that global and local processing are associated with distinct oscillatory patterns in the theta (4–8 Hz) and beta (12–25 Hz) frequency bands, respectively [98].

Neuroimaging and electrophysiology have also revealed how the PPC integrates visual information through the dorsal visual pathway. This pathway plays a pivotal role in visuospatial attention and action-oriented processes, often referred to as “vision-for-action” [99]. The PPC governs attentional allocation and ensures that visual information at both global and local levels is processed in a coordinated manner. Disruptions in this pathway, whether due to injury or experimental manipulations, often result in impaired integration of global and local features [79,100–102], as observed in patients with dorsal stream deficits [103–106].

Experimental studies using transcranial magnetic stimulation have provided causal evidence for hemispheric roles in global and local processing. For instance, Mevorach et al. [78] demonstrated that repetitive TMS (rTMS) applied to the left PPC selectively enhanced global-to-local interference, reinforcing the role of the left hemisphere in processing local details. Conversely, inhibition of the right PPC reduced interference effects, highlighting its essential role in facilitating global processing. Other studies have employed rhythmic TMS to manipulate neural oscillations directly. Romei et al. [79,100] showed that stimulating the PPC at theta frequency enhanced global processing, while beta-frequency stimulation favored local processing. These findings emphasize the role of neural oscillations in mediating the balance between global and local processing.

Nevertheless, inconsistencies persist across the existing body of work. For example, while TMS-induced modulations of global and local processing have been reported, the effects over the left PPC are less pronounced in some studies [100], raising questions about the robustness of these asymmetries. Romei et al. [79] demonstrated that 10 Hz alpha stimulation over the right PPC disrupted global processing significantly, while left PPC stimulation primarily affected local processing with a minor enhancement of global processing. In other words, transcranial magnetic stimulation has the potential to cause bias in both local and global processing. An important consideration is that some of these studies have employed an online TMS stimulation protocol to specific brain areas, applied just before the stimulus presentation to the subjects [79,100]. However, a few studies [78,107] applied rTMS stimulation over left and right PPC that were unable to have a major effect on both local and global processing.

Moreover, most studies in this domain have relied heavily on Navon stimuli to evaluate the neural correlates of global and local processing, leaving other tasks, such as those engaging holistic face perception, relatively underexplored. This reliance on hierarchical stimuli limits our understanding of whether similar neural mechanisms govern global-local processing across different cognitive domains. While rhythmic TMS has demonstrated its potential to bias processing orientations, the mechanisms underlying these effects, particularly in the context of holistic processing tasks like face perception, remain poorly defined.

To address these limitations, it is crucial to adopt methods that provide both precision and durability in manipulating processing orientations. Continuous theta-burst stimulation, an advanced TMS protocol, offers a promising approach. Unlike conventional rTMS, which delivers pulses at fixed frequencies, cTBS applies high-frequency bursts designed to induce long-term changes in cortical excitability, such as long-term depression (LTD) [108–111]. This protocol has been shown to produce more consistent and lasting effects on cortical networks, with studies demonstrating its ability to influence both behavioral and cognitive processes [112,113]. For instance, cTBS applied to the primary motor cortex (M1) induces durable reductions in synaptic excitability, suggesting its potential utility in modulating the PPC to bias global-local processing orientations effectively.

By incorporating cTBS into tasks involving both Navon stimuli and composite faces, the current study aims to overcome the limitations of previous approaches. This methodology provides an opportunity to examine how causal manipulations of PPC activity impact not only hierarchical visual processing but also the broader domain of holistic face perception, thereby bridging a critical gap in the literature.

### Objectives and rationale of the current study

While prior research has provided compelling evidence for hemispheric asymmetries and neural mechanisms underlying global and local processing, its applicability to broader cognitive contexts remains limited. A substantial body of work has focused on using the priming method to manipulate local and global processing orientations and assess their influence on subsequent tasks, particularly face recognition [24,25,75,76]. These studies have demonstrated that engaging in Navon tasks can bias participants’ performance on face recognition, with global priming typically enhancing face processing and local priming impairing it. However, despite their utility, priming methods exhibit significant limitations.

Priming effects are often transient, decaying rapidly and lacking the durability required for long-term investigations of cognitive processes [76,77,114]. Moreover, the precision of priming is insufficient for a thorough exploration of the causal roles of specific brain regions in modulating local and global processing. These constraints underscore the necessity for alternative techniques capable of inducing robust and lasting effects. Transcranial magnetic stimulation offers such a solution, as it allows for localized and controlled modulation of cortical excitability, providing a causal framework for studying the neural underpinnings of processing orientations [82,115,116].

Another significant limitation in previous studies is the underutilization of cognitive modeling to link neural mechanisms with behavioral outcomes. While neuroimaging and TMS studies provide invaluable insights into the neural substrates of global and local processing, they often lack a structured framework to quantify how these neural disruptions translate into cognitive processes, such as evidence accumulation. Cognitive modeling addresses this gap by linking neural activity to latent cognitive mechanisms, enabling a more comprehensive understanding of how hemispheric differences influence processing orientations. For instance, diffusion-based models or accumulator frameworks [117–119] can capture decision-making dynamics, revealing how disruptions in posterior parietal cortex activity shape task performance over time. Incorporating these models here allows for a deeper exploration of the interplay between hemispheric specialization and hierarchical visual processing.

To address these limitations, the current study employs continuous theta-burst stimulation, a TMS protocol known for its ability to produce durable after-effects on specific neural networks. Unlike traditional repetitive TMS, which requires higher intensities and longer durations of stimulation, cTBS applies brief, high-frequency bursts that mimic natural neuronal firing patterns, resulting in more efficient and safer modulation of cortical excitability [109,110]. By targeting the posterior parietal cortex using cTBS, this study aims to induce biases in local and global processing orientations across two complementary tasks: the hierarchical Navon task and the composite face task.

This dual-task approach offers a novel opportunity to bridge gaps in the literature. While most previous studies have focused on hierarchical stimuli, holistic face processing remains relatively underexplored in the context of global-local mechanisms. Both Navon stimuli and composite faces engage holistic processing to varying extents, yet it remains unclear whether the same neural mechanisms underlie these tasks. By incorporating both paradigms within a unified experimental framework, the current study seeks to investigate whether hemispheric differences observed in hierarchical tasks extend to holistic face perception. We hypothesize that any disruptions in global or local processing observed with Navon letters will similarly manifest in composite face tasks, supporting the notion of shared holistic processing mechanisms.

To test these hypotheses, participants completed a hierarchical Navon task both before and after TMS stimulation, enabling the measurement of any changes in selective attention to global or local levels. Following this, participants performed a composite face task to assess the influence of TMS-induced biases on holistic face processing. We predicted that applying cTBS over the right PPC would impair global processing with a minor trend toward enhancing local processing, while stimulation over the left PPC would disrupt local processing with a slight tendency to enhance global processing. The inclusion of a sham condition allowed for direct comparisons to determine the specific effects of cTBS.

Another distinguishing feature of the current study is its integration of cognitive modeling with neurostimulation methods. By employing the Diffusion Model of Conflict [120], we provide a computational framework to quantify how cTBS-induced biases on global and local processing affect decision-making dynamics across both tasks. This computational approach not only complements behavioral analyses but also allows us to quantify how experimental manipulations influence decision-making over time, linking behavioral outcomes to the underlying neural mechanisms modulated by cTBS. Furthermore, cognitive modeling enables a more nuanced interpretation of task-specific effects, offering insights that extend beyond traditional behavioral analyses.

By employing these innovative methods, this study advances our understanding of the neural mechanisms underlying global and local processing and their relationship to hemispheric specialization. Moreover, this research provides new insights into how TMS-induced manipulations can generalize across cognitive domains, offering a more integrative perspective on visual processing. The findings have the potential to inform therapeutic interventions for disorders characterized by impairments in visual perception and attention, such as autism spectrum disorder, visual agnosia, and prosopagnosia.

## Materials and methods

### Participants and ethics

A total of 36 right-handed postgraduate students from Shahid Beheshti University, aged between 18 and 30 years, participated in this study. All participants had normal or corrected-to-normal vision, confirmed via the confrontation method to exclude visual field defects. Additionally, all participants provided written informed consent after receiving detailed information about the experimental procedures. The research protocol was approved by the ethical committee of Shahid Beheshti University, with the ethics code IR.SBU.REC.1399.006; Date: 2020-02-22. No participants under the age of 18 were included. Participant recruitment occurred between October 15, 2020 and February 20, 2021. Demographic metadata (e.g., gender) are accessible via the project’s open-source framework.

Eligibility for participation was determined based on stringent inclusion and exclusion criteria. Inclusion criteria required participants to be within the specified age range, possess normal or corrected vision, and be able to understand and sign the informed consent form. Exclusion criteria adhered to established safety guidelines for TMS, ensuring participants had no history of epilepsy, psychiatric or neurological disorders, significant head trauma, or drug and alcohol abuse [121,122]. Furthermore, individuals with a history of tricyclic antidepressant or psychotic medication use, head or spinal surgery, or the presence of metallic implants such as cardiac pacemakers (excluding dental fillings) were excluded [109].

The experimental tasks were displayed on 17-inch monitors with a resolution of 1920 × 1080 pixels, positioned 60 cm from the participants. Responses to all tasks were made via a computer keyboard, with participants instructed to respond as quickly and accurately as possible. Practice trials were conducted to familiarize participants with the task requirements. Participants who demonstrated issues such as consistent misinterpretation of task instructions or extreme error rates during practice sessions were excluded. The students were informed prior to participation that the experiment’s data could be used in future publications anonymously. Finally, financial compensation was provided to ensure voluntary participation.

### Inclusivity in global research

Additional information regarding the ethical, cultural, and scientific considerations specific to inclusivity in global research is included in the Supporting Information (S1 Checklist).

### Behvaioral tasks

This study employed two well-established behavioral tasks to investigate global and local processing: Navon’s paradigm and the composite face task. These tasks were selected to assess hierarchical and holistic processing, providing complementary insights into cognitive mechanisms.

### Navon’s paradigm

**Fig 1:**
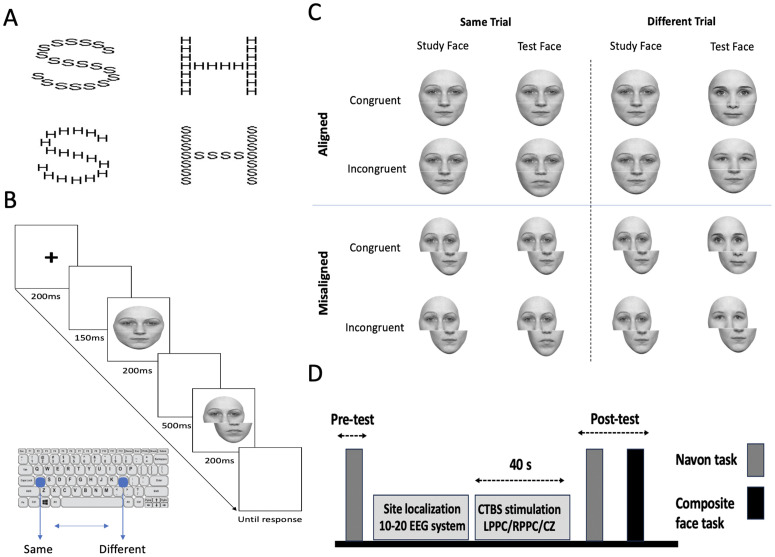
Experimental task design. **(A)** Examples of hierarchical Navon stimuli used in this study, illustrating both congruent (e.g., a large ‘H’ made of smaller ‘H’s) and incongruent (e.g., a large ‘H’ composed of smaller ‘S’s) conditions. **(B)** A schematic of a single misaligned composite face trial, showing the sequence of events. Study faces were consistently presented in the aligned condition, while test faces were displayed in either aligned or misaligned configurations. **(C)** Illustration of the complete composite face paradigm, including congruency and alignment manipulations, designed to assess holistic processing through congruent-aligned, congruent-misaligned, incongruent-aligned, and incongruent-misaligned conditions. **(D)** Overview of the experimental timeline. Each participant completed three sessions involving cTBS: left PPC stimulation, right PPC stimulation, and sham stimulation. The sessions were counterbalanced across participants following a Latin square design, ensuring at least a 24-hour gap between sessions to prevent carryover effects. **Note.** The face images shown are standardized stimuli from the face-categorization-lab dataset and do not depict any participant from the current study.

Navon stimuli consisted of large letters, either ‘H’ or ‘S’, constructed from smaller letters, either ‘H’s or ‘S’s, as adapted from Gerlach and Poirel [123] (Fig 1). Participants completed four experimental blocks, two of which required identifying the global letter (global attentional blocks), while the remaining two focused on the local letter (local attentional blocks). The order of global and local blocks was counterbalanced using a Latin square design, ensuring six possible sequences. Half of the participants began with global identity judgments.

The large letters measured 208 pixels in width and 340 pixels in height, while the small letters were 34 pixels wide and 42 pixels high. A central fixation cross measuring 1 cm by 1 cm appeared before each stimulus presentation. All stimuli were black and displayed against a white background on a computer monitor.

Each block comprised 48 trials, evenly split into 24 congruent and 24 incongruent trials. In congruent trials, the letter identity was the same at both global and local levels (e.g., a large ‘H’ composed of smaller ‘H’s), while in incongruent trials, the global and local letters differed (e.g., a large ‘H’ made of smaller ‘S’s). Stimuli were presented randomly at one of four screen locations—left, right, top, or bottom—relative to the fixation point to prevent participants from adopting a local bias by fixating on a specific part of the global letter [124,125]. The congruency order was also randomized, with an equal number of congruent and incongruent stimuli (N = 12, 6 congruent, and 6 incongruent) presented at each location per block. The center of the global letter was positioned 6 cm from the fixation cross.

Each trial began with a fixation cross displayed centrally for 100 ms, during which participants were instructed to focus their gaze. Afterward, the experimental stimulus appeared for 300 ms, followed by a 4-second response window. Participants were required to respond as quickly and accurately as possible by pressing the appropriate keys on a keyboard. A fixation cross was displayed between trials to maintain central fixation.

Before beginning the main experiment, participants completed 16 practice trials, which included feedback to reinforce task comprehension. Correct responses, defined as accurately identifying the target letter at the instructed level (global or local), were indicated with a green marker, while incorrect responses were highlighted in red. During the main experiment, no feedback was provided to avoid influencing response patterns.

Both accuracy and response times (RTs) were recorded. Accuracy was calculated as the percentage of correct responses out of the total number of trials within each condition (e.g., congruent and incongruent trials). Response times were measured from the onset of the experimental stimulus to the participant’s keypress, capturing the time required to process and respond to the target letter.

### Composite face task

The composite face task evaluated holistic face processing using ten grayscale, full-front photographs of Caucasian faces (five male and five female) with neutral expressions, selected from the face-categorization-lab dataset [47]. To ensure consistency, all faces were standardized for picture height, converted to grayscale, and equalized for mean pixel luminance. External features, such as hair, glasses, and ears, were removed to isolate internal facial features. Each face was horizontally bisected to create composite stimuli by pairing the top half of one individual’s face with the bottom halves of four different individuals of the same sex. This process generated 40 aligned and 40 misaligned composite faces, resulting in a total of 80 stimuli.

Misaligned faces were created by shifting the bottom half of the face horizontally to the right, such that the edge of the top half aligned with the center of the bottom half [70]. A 3-pixel white line was placed between the top and bottom halves to clearly separate them and facilitate selective attention to the target region [43,55]. In the aligned condition, each stimulus measured 256 × 259 pixels, whereas misaligned faces measured 256 × 320 pixels.

Each trial (see Fig 1) began with a fixation cross displayed for 200 ms, followed by a white blank screen for 150 ms. Subsequently, the study face was presented for 200 ms, followed by another white blank screen for 500 ms. Finally, the test face appeared for 200 ms, after which a blank screen remained visible until the participant provided a response. The inter-trial interval was 1000 ms. Participants were instructed to focus solely on the top half of the composite face and decide whether it matched the top half of the study face, while ignoring the bottom halves.

The experiment included 160 trials, divided into four blocks. Each block contained ten trials for each of the four conditions: congruent-aligned, congruent-misaligned, incongruent-aligned, and incongruent-misaligned. In aligned trials, the two halves of the test face were presented in alignment. In misaligned trials, the study face remained aligned while the test face was misaligned. Participants responded by pressing one of two designated keys: one for “same” and the other for “different.” To ensure consistent and unbiased responses, participants were instructed to use one key with their right hand and another key with their left hand (e.g., pressing “1” for “same” and “2” for “different” across all conditions). This design required the involvement of both hands, promoting balanced motor engagement across trials. The counterbalance method was utilized to assign response keys, ensuring that half of the participants used one configuration, while the other half used the opposite configuration.

Both accuracy and RTs were recorded to evaluate performance. Accuracy was calculated as the percentage of correct responses, where participants accurately identified whether the top halves of the study and test faces were the same or different. This measure was computed separately for each of the four experimental conditions: congruent-aligned, congruent-misaligned, incongruent-aligned, and incongruent-misaligned trials. Response times were measured as the interval between the onset of the test face and the participant’s keypress. This allowed the study to capture the duration required for perceptual processing and decision-making specific to the composite face task.

Before the experimental phase, participants completed 16 practice trials to familiarize themselves with the task and its requirements. Feedback was provided during these trials to correct errors and ensure adherence to the instructions. Participants were allowed short breaks between blocks during the main experiment to minimize fatigue.

### Brain stimulation and experimental procedures

A continuous theta-burst stimulation protocol [109] was applied to disrupt cortical activity at the target locations. The protocol involved delivering three-pulse bursts at 50 Hz, repeated every 200 ms for a total duration of 40 seconds, resulting in 600 pulses. Stimulation intensity was set at 8% of the participant’s resting motor threshold (RMT). The RMT was recalculated at the start of each session as the lowest stimulator intensity capable of eliciting a visible twitch in the first dorsal interosseous (FDI) muscle of the contralateral hand in at least 5 out of 10 single-pulse TMS trials. Previous studies have demonstrated high concordance between electromyographic recordings and visible twitch methods for determining RMT, with the visible twitch method being widely adopted for its practicality and reliability [109,126–130].

Continuous theta-burst stimulation was delivered using a Super Magstim Rapid2 magnetic stimulator (Magstim, Whiteland, UK) connected to a figure-of-eight coil with a diameter of 70 mm (Double 70 mm Air Film Coil). The coil was positioned tangentially to the scalp at a ~45° angle from the anterior-posterior midline, with the handle pointing ventrally. This specific coil was chosen due to its design, which includes an internal cooling fan to prevent overheating during stimulation, thereby reducing potential side effects.

**Fig 2:**
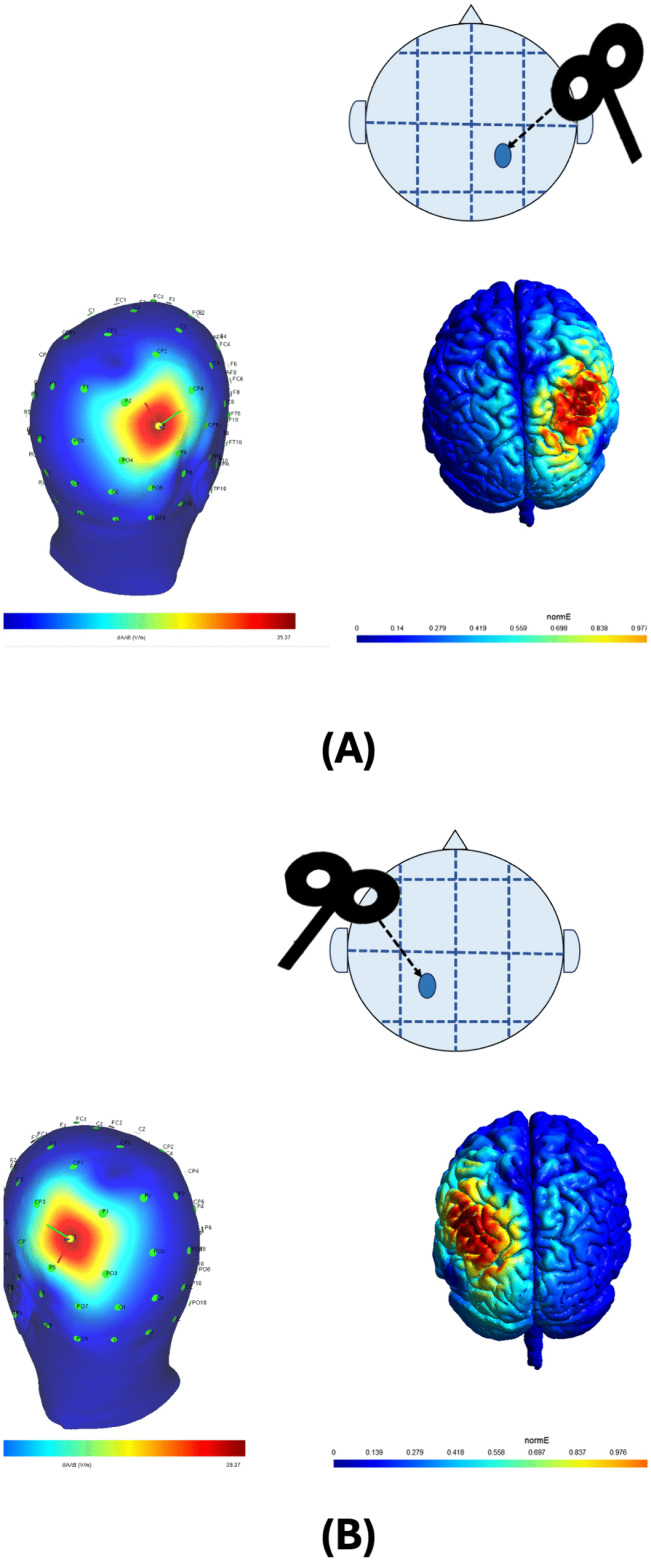
TMS coil positioning and stimulation sites. **(A)** Configuration of the figure-of-eight TMS coil during stimulation over the P3 region. The upper image shows the positioning of the coil over the identified “hot spot” (blue circle) on the P3 region, while the subsequent images depict the stimulation sites targeted for cTBS application over the P3 region. **(B)** Configuration of the figure-of-eight TMS coil during stimulation over the P4 region. The upper image illustrates the positioning of the coil over the identified “hot spot” (blue circle) on the P4 region, and the subsequent images represent the stimulation sites targeted for cTBS application over the P3 region. The locations of the left and right PPC were determined using the 10–20 EEG electrode placement system, ensuring precise targeting of cortical regions involved in attentional and perceptual processing.

After determining the RMT, cTBS was applied to P3 and P4 electrode sites, corresponding to the left and right posterior parietal cortex, respectively, as per the 10–20 electrode position system of the American Electroencephalographic Association (1994). These regions overlay the intraparietal sulcus and were targeted based on prior studies implicating their role in attentional and perceptual processing [131]. For the sham condition, stimulation was applied over the Cz electrode site with the coil placed perpendicular to the scalp to minimize effective stimulation. Additionally, the stimulation intensity for the sham condition was reduced to 40% of the participant’s RMT, mimicking the auditory and tactile sensations of active stimulation without inducing significant cortical effects [132,133]. A schematic representation of the site localization is shown in Fig 2.

Participants underwent all three stimulation conditions (left PPC, right PPC, and sham) in a counterbalanced crossover design, with the order of stimulation randomized across individuals according to a Latin square design. To prevent carryover effects, experimental sessions were separated by at least 24 hours, as the effects of cTBS are probably known to dissipate within this timeframe [134]. Consequently, each participant completed three sessions over a minimum of three days. An overview of the session protocol, including the sequence of stimulation and behavioral tasks, is presented in Fig 1.

### Diffusion model of conflict

Resolving cognitive conflict requires understanding how task-relevant and task-irrelevant information compete during decision-making. Computational models play a vital role in explaining these dynamics. The diffusion model, a cornerstone of decision-making research, offers a mechanistic framework for understanding how individuals resolve conflicts between competing cognitive processes. Originally developed to model simple two-choice decisions [39,135], this model has been extended to capture the dynamics of conflict tasks, where task-relevant and task-irrelevant stimuli influence response selection. Three prominent computational models have been developed to account for conflict processing in decision-making tasks, each incorporating task-relevant and task-irrelevant information sources. These models include the dual-stage two-phase model (DSTP) [136], the shrinking spotlight model (SSP) [137], and the diffusion model for conflict tasks (DMC) [120]. These models capture different temporal effects of irrelevant information on decision processes. The DSTP considers irrelevant processes to have an effect in an all-or-non manner in the time course of the decision process, maintaining some constant effect till some time point and then vanishing after that. In contrast, both the SSP and DMC propose that the impact of irrelevant processes evolves gradually throughout the decision-making process.

The distinctive property of the DMC relative to the other two models is that it assumes that the total effect of the irrelevant process diminishes over the course of the decision, meaning that the earlier effects are compensated later in the decision process. This allows the model to predict, among other trends, a decreasing difference in the response time quantiles of two congruency conditions where later response times are similar but earlier ones are different [120]. This trend is represented by a negative-going delta function, which plots the difference between response time quantiles for incongruent and congruent conditions against the mean of these values [138]. This is a representative of the time course of the congruency effect. In our data, there is an evident trend of this kind, which makes it reasonable to use the DMC.

Central to the DMC framework is the concept of superimposed diffusion processes, where task-relevant (controlled) and task-irrelevant (automatic) information are simultaneously accumulated over time to reach a decision threshold. Controlled processes typically guide the response toward task goals, while automatic processes may introduce interference, particularly in incongruent trials where competing stimulus features activate conflicting responses. The decision variable represents the relative evidence of two choices gathered thus far. The interaction of these processes can be mathematically described as a combined Wiener diffusion process, where the total drift rate reflects the summation of controlled and automatic components [120]. The dynamics of the decision process are modeled using the following stochastic differential equation:


$$dX_{t}=[\mu_{r}+\mu_{i}(t)]dt+\sigma dW_{t},$$

where *t* denotes time, $X_{t}$ is the time-dependent state of the decision, $\mu_{r}$ represents the constant drift rate of the relevant process, $\mu_{i}$ is the time-dependent drift rate of the irrelevant process, σ is the diffusion constant, and $W_{t}$ is a standard Wiener process. The drift of the irrelevant process is the time derivative of the mean state of the irrelevant process, which is considered to follow a scaled gamma density function:


$$Ae^{-t/\tau }{\left[\frac{te}{(a-1)\tau }\right]}^{a-1}.$$

This function has a right skewed unimodal shape which reached its maximum value of *A* in time (a−1tau). The *a* controls the shape of the function and τ scales the time. When the decision variable reaches a subjective level of choice conformance, the decision is made. The response time is the time to reach the criterion plus the non-decision time, including stimulus encoding and motor execution. The parameters of the model are:

**drc:** mean value of the relevant process contribution to decision formation per unit time ($\mu_{r}$ in the above formula).
**amp:** maximum mean effect of the irrelevant process on the decision variable (*A* in the above formula).
**tau:** time scale of the mean effect of the irrelevant process on the decision variable (τ in the above formula).
**aa_shape:** shape parameter of the mean effect of the irrelevant process on the decision variable (*a* in the above formula).
**sigma:** diffusion constant of the random process of decision variable (σ in the above formula).
**bnds:** boundary representing the satisfactory level of choice conformation.
**strp:** relative proximity of the starting point of the decision process to the boundaries of different responses.
**res:** residual time of response representing time consumed by non-decisional processes.

**Fig 3:**
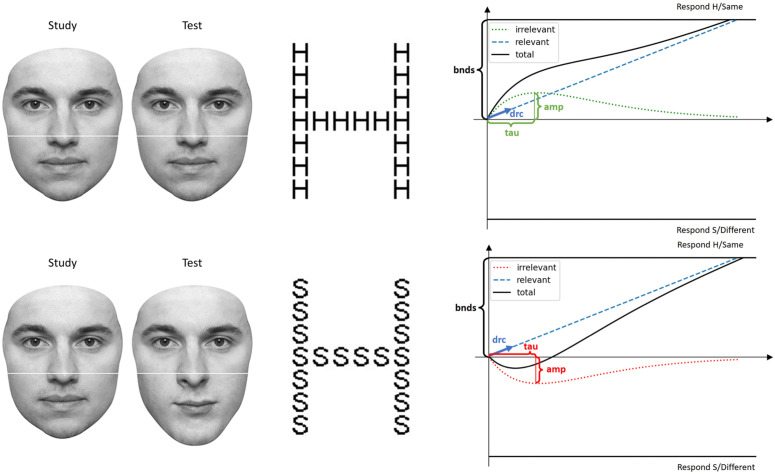
Simulated diffusion model trajectories for congruent and incongruent conditions. The mean state of the diffusion process for congruent (top) and incongruent (bottom) conditions, modeled for a sample of two tasks: the Navon task in the global condition, where participants identify the global letter of the stimulus, and the composite face task in the aligned condition, where participants decide whether the top half of the face has changed. The shapes of the diffusion trajectories are determined by the parameter values: amp=20 (amplitude of the irrelevant process), tau=30 (time scale of the irrelevant process), aa_shape=2 (shape parameter of the Gamma distribution), drc=0.5 (drift rate of the relevant process), strp=0 (starting point of the decision variable), and bnds=75 (decision boundaries). The time to maximum irrelevant interference, mathematically defined as (aa_shape−1textittau), simplifies to tau  because aa_shape  is fixed at 2. The figure captures the temporal evolution of the decision variable under each condition, highlighting the transient peak of irrelevant information and its subsequent decline, which align with the theoretical dynamics proposed by the DMC. **Note.** The face images shown are standardized stimuli from the face-categorization-lab dataset and do not depict any participant from the current study.

In this study, the Navon task and the composite face task were modeled using the DMC framework. In the Navon task, the relevant process in the local (global) conditions corresponds to the local (global) process of the stimulus identity and the global (local) process is the irrelevant process. In the composite face task, the relevant process is the process of the top half of the face stimulus, while the process of the bottom half is the irrelevant process. The application of the DMC model to both tasks is illustrated in Fig 3.

### Model fitting procedure

The model was individually fitted to each condition of the experiment, covering the dimensions of stimulation type, level, congruency, and time for the Navon task, as well as stimulation type, alignment, and congruency for the composite face task. This approach ensured a detailed representation of the effects under various experimental manipulations. Although behavioral differences were observed between response alternatives (e.g., “S/H” in the Navon task and “Same/Different” in the composite face task) at the individual level, no statistically significant differences emerged at the population level. Specifically, for the Navon task, accuracy yielded F(1,34)=2.43, p=0.13, and response time yielded F(1,34)=1.05, p=0.31. For the composite face task, accuracy yielded F(1,32)=1.28, p=0.27, and response time yielded F(1,32)=2.23, p=0.15. These observations, along with the necessity for sufficient dataset size during model fitting, justified pooling data across response alternatives, with accuracy serving as the modeled outcome rather than response alternatives.

Model fitting was achieved by minimizing an adapted chi-squared cost function [139]:


$$\chi^{2}=\sum_{i=1}^{2}N_{i}\sum_{j=1}^{m}\frac{p_{ij}-\pi_{ij}}{\pi_{ij}},$$

where $N_{i}$ represents the number of data points for the two congruency conditions, and $p_{ij}$ and $\pi_{ij}$ denote the observed and predicted proportions of data points in each bin of correct and error response times, respectively. The bins (*m*) were determined using the experimental data response time quantiles adaptively based on the number of observations. Quantiles were used for binning as follows:(0.1, 0.3, 0.5, 0.7, 0.9) for trials numbering 30 or more,(0.3, 0.5, 0.9) for 15–29 trials, (0.5) for 6–14 trials, and a single bin for fewer than six trials.

Predicted values were derived from simulations using a 1 ms time step, with 50,000 simulated trials per compatibility condition. Differential evolution was employed to optimize the parameters of the model by minimizing the cost function. The diffusion coefficient as a scaling parameter was fixed at 4 to ensure consistent scaling of the decision process. Following the recommendations of [139], the gamma distribution’s shape parameter was fixed at 2, improving the recovery of other model parameters. Additionally, the decision starting point was set midway between the decision boundaries to reflect accuracy-based coding in the model.

**Fig 4:**
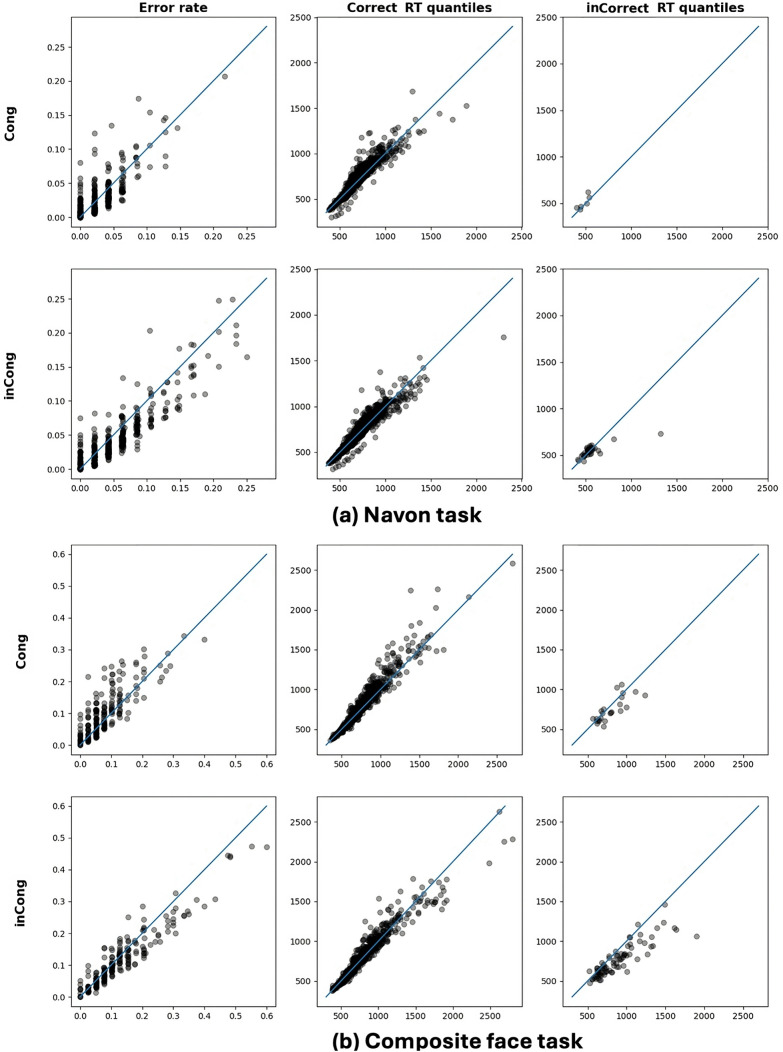
Model fit: predicted vs. actual behavioral data. Comparison of predicted and actual values of behavioral data across experimental conditions, where the horizontal axis represents the actual values of behavioral data and the vertical axis represents the predicted values. Each data point represents the predicted versus actual values for error rates (left), correct response time quantiles (middle), and error response time quantiles (right). Panel (a) corresponds to the Navon task, with conditions of time, stimulation, and level stacked within the plots. Panel (b) corresponds to the composite face task, with conditions of stimulation and alignment stacked. Rows indicate congruency conditions (congruent vs. incongruent), highlighting the model’s ability to accurately capture behavioral patterns across tasks and conditions.

Parameter recovery under the described fitting routine demonstrated reliability, with performance comparable to or better than prior work [139,140], particularly in scenarios with reduced dataset sizes (e.g., 50 trials). The results of the model fitting procedure are illustrated in Fig 4, which compares predicted versus observed values for error rates and response time quantiles across all conditions. Note that the time scale of the model was standardized to 1 ms, and corresponding parameter estimates were appropriately scaled. The implementation utilized Python’s pydmc library (https://github.com/igmmgi/pydmc) of Mackenzie and Dudschig [141], with some modifications and implementation of the adapted chi-squared cost function used in White et al. [139].

### Statistical analyses

As the primary outcome, mean accuracy and response times for both the global/local and holistic face processing data were analyzed under active and sham conditions. Data preprocessing steps ensured the integrity of the analysis by excluding trials and blocks with anomalies. Specifically, trials with response times shorter than 300 ms were removed, as were blocks with accuracy levels below chance, determined using a binomial test with a *p*-value of 0.05. Additionally, blocks with unusually low accuracy, suggesting misinterpretation of instructions or key assignments, were excluded. These criteria resulted in the removal of 0.71% of Navon task data. Also, subject 23 was excluded entirely due to excessive missed trials, low accuracy, and prolonged response times.

In the composite face task, similar exclusion criteria were applied: trials with response times below 300 ms and blocks with sub-chance accuracy were removed, leading to the exclusion of 1.77% of the data. Subjects 9 and 10 were excluded for low accuracy, while Subject 4 was excluded due to corrupted data in the left posterior parietal cortex condition. Outliers from both Navon and composite face tasks were systematically identified and excluded based on the interquartile range (IQR), with values exceeding [Q1−1.5×IQR,Q3+1.5×IQR], where *Q1* and *Q3* represent the first and third quartiles, respectively.

To evaluate the effects of cTBS on task performance, repeated-measures analyses of variance (ANOVAs) were conducted independently for accuracy and response times from Navon and composite face tasks. These analyses assessed whether significant changes in performance metrics were driven by stimulation. Post hoc paired-samples *t*-tests were used to compare differences in both tasks between experimental and sham conditions, as well as pre-test versus post-test outcomes in the Navon task for specific stimulation sites.

Additionally, ANOVAs were performed within the framework of the DMC to investigate task condition effects at the parameter level. Pairwise comparisons were used to evaluate differences across DMC model parameters, providing insights into the cognitive mechanisms influenced by stimulation.

All statistical analyses were conducted using R (version 3.6.1) and the DMC modeling analysis [141], adhering to principles of transparency and reproducibility. For all comparisons, a significance level of p<0.05 was applied. This analytical approach provided a robust framework for assessing the influence of cTBS on hierarchical and holistic processing mechanisms.

## Results

### Performance in hierarchical Navon task

**Fig 5:**
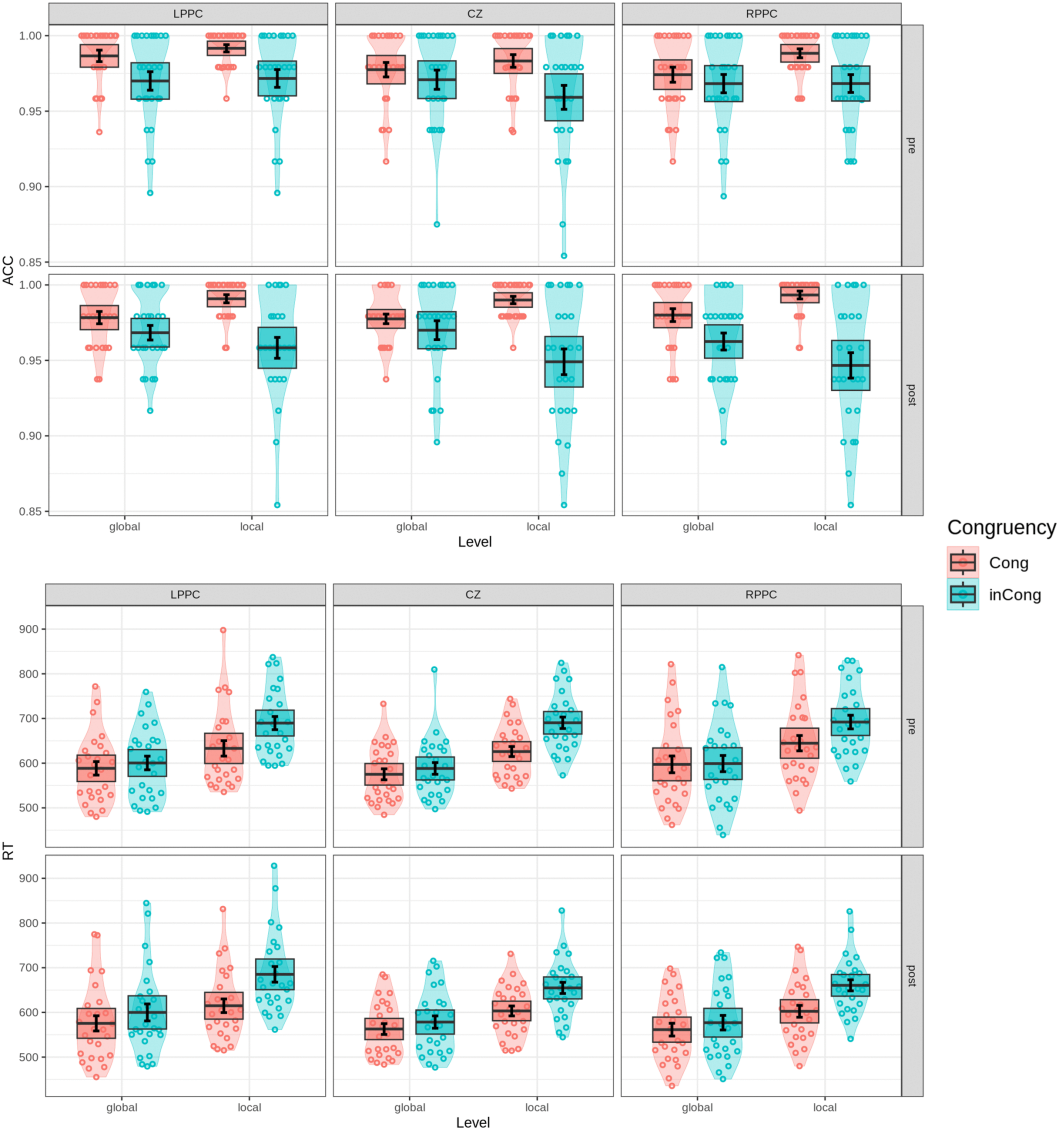
Behavioral performance in the Navon task. Violin plots with overlaid data points depict accuracy (top) and response times (RTs, bottom) across conditions. Error bars indicate one standard error of the mean, while shaded boxes represent the 95% confidence interval, assuming a hypothetical normal distribution of the mean. For the Navon task, conditions of congruency (congruent vs. incongruent) and level (global vs. local) are compared.

Repeated-measures ANOVA was conducted on participants’ response times and accuracy to assess the effects of Congruency (congruent, incongruent), Level (global, local), Time (pre-test, post-test), and Stimulation site (LPPC, RPPC, CZ). Analysis revealed a significant main effect of Level (F(1,25)=142.567, p<0.0001, $\eta_{G}^{2}=0.163$), with faster responses at the global level compared to the local level, reflecting a robust global precedence effect. Additionally, a main effect of Congruency was observed (F(1,25)=268.098, p<0.0001, $\eta_{G}^{2}=0.054$), with congruent trials eliciting faster judgments than incongruent trials. Importantly, a significant Level × Congruency interaction (F(1,25)=100.369, p<0.0001, $\eta_{G}^{2}=0.021$) indicated that the congruency effect was more pronounced for local trials than for global ones, a pattern depicted in Fig 5.

In the error rate analysis, there was no significant main effect of Level when comparing global and local conditions (F(1,24)=0.053, p=0.819). However, a significant main effect of Congruency was observed (F(1,24)=48.299, p<0.0001, $\eta_{G}^{2}=0.134$), with participants demonstrating higher accuracy in congruent trials than in incongruent ones. Moreover, a significant Level × Congruency interaction (F(1,24)=11.736, p<0.005, $\eta_{G}^{2}=0.035$) mirrored the RT pattern, indicating greater interference from global-to-local processing (F(1,24)=21.601, p<0.0001, $\eta_{G}^{2}=0.043$) than from local-to-global processing (F(1,24)=31.963, p<0.0001, $\eta_{G}^{2}=0.238$). Fig 5 provides details on mean accuracy for each of the four conditions. Taken together, these behavioral findings underscore the dominance of global processing over local processing, consistent with previous research on global precedence effects [3].

To evaluate whether cTBS stimulation as a manipulating method could bias global or local processing, a 2×3×2×2 repeated-measures ANOVA (Time × Stim × Level × Congruency) was performed for both RTs and accuracy. Accuracy analysis revealed no significant main effect of either Time (F(1,24)=3.249, p=0.084) or Stim (F(2,48)=2.156, p=0.127), suggesting that mean accuracy was comparable between pre-test and post-test conditions across LPPC, RPPC, and CZ stimulation sites. The Time × Stim interaction was also non-significant (F(2,48)=0.858, p=0.43). However, a significant Congruency × Time interaction (F(1,24)=5.62, p=0.026, $\eta_{G}^{2}=0.009$) emerged, with post hoc analyses indicating that the congruency effect was significantly larger in the post-test (F(1,24)=51.471, p<0.0001, $\eta_{G}^{2}=0.192$) compared to the pre-test (F(1,24)=17.023, p<0.0001, $\eta_{G}^{2}=0.081$). Furthermore, a significant Congruency × Level × Time interaction (F(1,24)=14.033, p<0.001, $\eta_{G}^{2}=0.006$) was observed, with post hoc analyses showing that global-to-local interference was more pronounced in the post-test than in the pre-test. These results suggest that cTBS stimulation did not significantly modulate local or global processing accuracy.

The RT analysis confirmed patterns observed in the accuracy data, showing no significant main effect of Stim (F(2,50)=1.008, p=0.372) or Stim × Time interaction (F(2,50)=2.170, p=0.125). However, unlike the accuracy analysis, the main effect of Time (F(1,25)=26.489, p<0.0001, $\eta_{G}^{2}=0.019$) was significant, with response times decreasing in post-test conditions compared to pre-tests. Pairwise comparisons between LPPC, RPPC, and CZ stimulation sites revealed non-significant differences in pre-test conditions. However, in post-test conditions, significant differences emerged for LPPC stimulation compared to CZ stimulation ($p_{\text{adj}}<0.05$, $\eta^{2}=0.439$) and LPPC compared to RPPC stimulation in local incongruent conditions ($p_{\text{adj}}<0.05$, $\eta^{2}=0.518$). Other pairwise comparisons were not statistically significant.

In summary, these results indicate a weak effect of cTBS stimulation on RTs but provide no substantial evidence of its impact on accuracy. The findings suggest that cTBS stimulation over the left and right posterior parietal cortex does not induce a consistent and noticeable bias in global or local processing through the lens of behavioral analysis.

### Performance in composite face task

**Fig 6:**
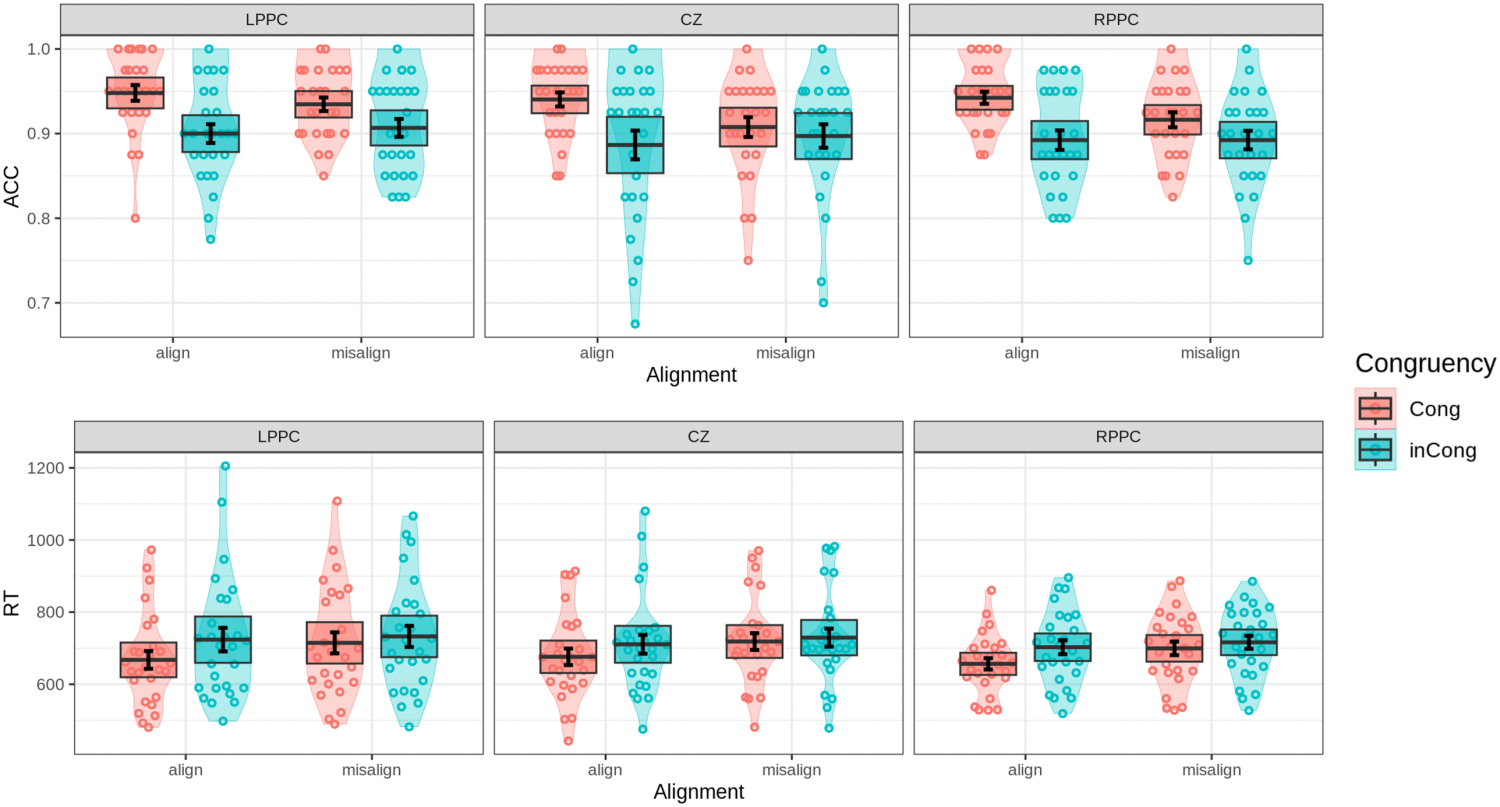
Behavioral performance in the composite face task. Violin plots with overlaid data points depict accuracy (top) and response times (RTs, bottom) across conditions. Error bars indicate one standard error of the mean, while shaded boxes represent the 95% confidence interval, assuming a hypothetical normal distribution of the mean. In the composite face task, results are organized by congruency and alignment (aligned vs. misaligned), reflecting the holistic interference effects.

To evaluate the effects of alignment, congruency, and stimulation on holistic face processing, a three-way repeated-measures ANOVA was conducted with Alignment (aligned, misaligned), Congruency (congruent, incongruent), and Stim (LPPC, RPPC, CZ) as factors. The analysis of accuracy revealed no significant main effect of Alignment (F(1,25)=2.647, p=0.116). However, a significant main effect of Congruency was observed (F(1,25)=24.000, p<0.0001, $\eta_{G}^{2}=0.095$), indicating lower accuracy in incongruent trials compared to congruent trials. The Alignment × Congruency interaction was also significant (F(1,25)=6.619, p<0.05, $\eta_{G}^{2}=0.018$), suggesting that alignment modulates the congruency effect. Specifically, in aligned conditions, participants were significantly less accurate when matching identical top halves of composite faces paired with different bottom halves (F(1,25)=25.759, p<0.0001, $\eta_{G}^{2}=0.17$) compared to misaligned conditions (F(1,25)=5.866, p<0.05, $\eta_{G}^{2}=0.036$). This interaction, shown in Fig 6, reflects the influence of holistic processing, wherein alignment amplifies interference from irrelevant bottom halves.

The RT analysis corroborated the patterns observed in accuracy. Significant main effects of both Congruency (F(1,26)=52.096, p<0.0001, $\eta_{G}^{2}=0.015$) and Alignment (F(1,26)=36.833, p<0.0001, $\eta_{G}^{2}=0.013$) were observed, with faster responses in aligned trials compared to misaligned ones. A significant Alignment × Congruency interaction (F(1,26)=7.043, p<0.05, $\eta_{G}^{2}=0.004$) further highlighted the interference effect of the bottom halves in aligned trials, underscoring the robust tendency for holistic processing of faces.

The analysis of cTBS stimulation effects on holistic face processing yielded non-significant main effects on both accuracy (F(2,50)=2.932, p=0.063) and RT (F(2,52)=0.387, p=0.681). These findings suggest that overall performance remained consistent across LPPC, RPPC, and CZ stimulation conditions. Pairwise comparisons indicated that only the mean differences in accuracy between LPPC and CZ stimulation, as well as between LPPC and RPPC in congruent misaligned trials, were statistically significant ($p_{\text{adj}}<0.05$, $\eta^{2}=4.751$ and $p_{\text{adj}}<0.05$, $\eta^{2}=4.861$, respectively).

The three-way interaction among Stim, Alignment, and Congruency was non-significant for both accuracy (F(2,50)=0.680, p=0.511) and RT (F(2,52)=0.225, p=0.799). Similarly, no significant interactions were observed between Stim × Congruency or Stim × Alignment. These results indicate no compelling evidence that cTBS stimulation altered holistic face processing performance.

In summary, the findings demonstrate robust holistic interference effects under aligned conditions, as evidenced by the congruency-by-alignment interaction. However, cTBS stimulation did not significantly modulate participants’ performance, suggesting that stimulation over the posterior parietal cortex does not exert a consistent influence on the integration of global and local facial features.

### Cognitive modeling results

#### Navon task.

**Fig 7:**
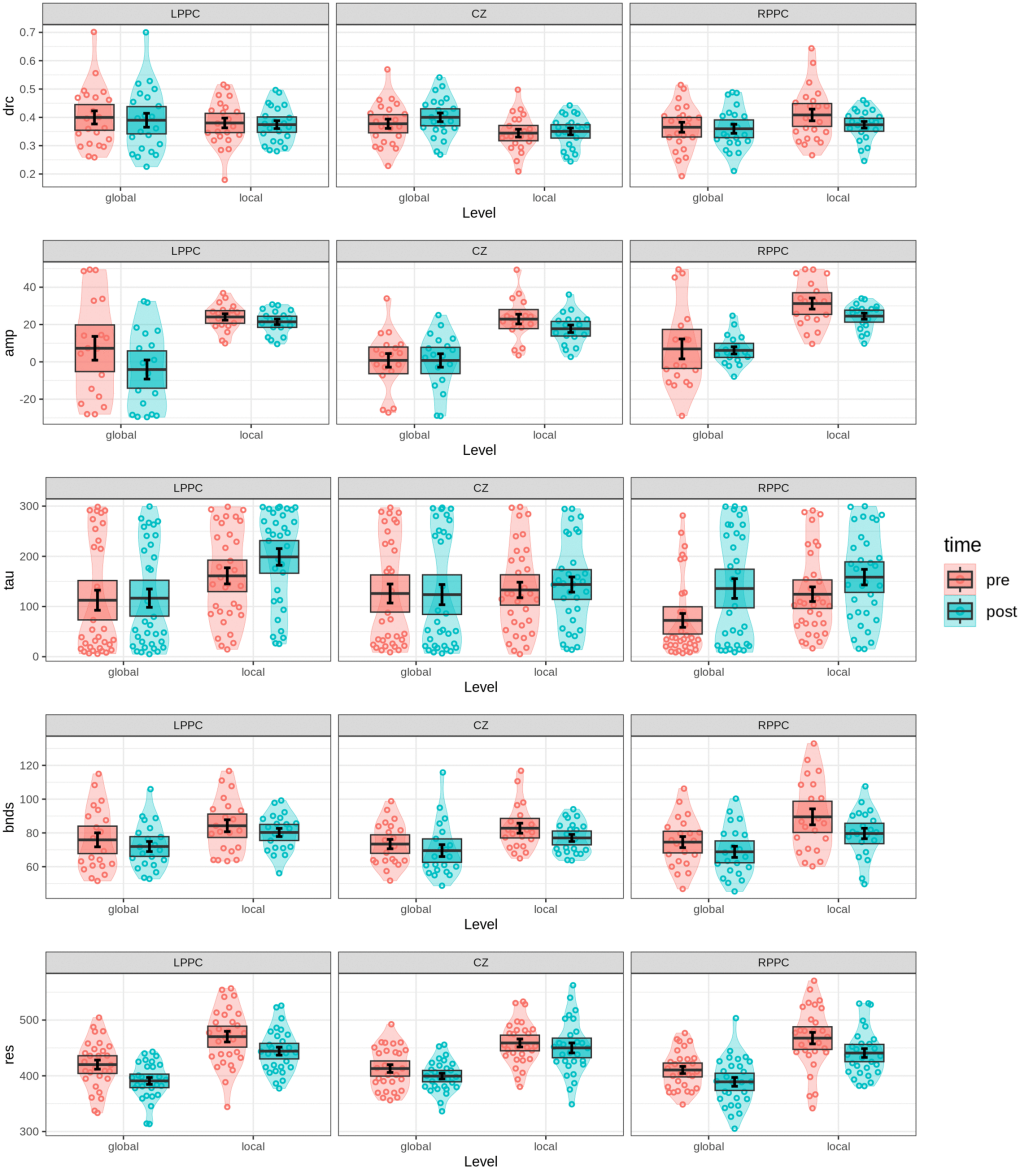
Parameter values of the DMC model for the Navon task. Violin plots illustrate the distributions of key parameters—*drc* (drift rate), *amp* (amplitude),*tau* (latency), *bnds* (decision boundary), and *res* (residual time)—across distinct stimulation (LPPC, RPPC, CZ), Level (global, local), and Time (pre-test, post-test) conditions. Overlaid data points reflect individual participant values, while error bars indicate one standard error around the mean. Shaded boxes represent the 95% confidence intervals assuming a normal distribution for the mean. These visualizations highlight variations in cognitive processing dynamics under different experimental conditions.

To assess the effects of cTBS stimulation and task conditions on the cognitive mechanisms underlying global and local processing, ANOVAs were conducted on the key parameters of the DMC. These parameters capture the dynamics of decision-making, automatic activation, decision thresholds, and non-decision processes under conditions of congruency, level (global vs. local), and stimulation site. The amplitude parameter, which captures the peak effect of automatic activation during decision-making, demonstrated a significant main effect of Level, see Fig 7. As expected, the amplitude was significantly lower in global trials compared to local trials (F(1,17)=122.361, p<0.0001, $\eta_{G}^{2}=0.33$), highlighting stronger automatic activation for local processing. This effect was consistently observed across stimulation conditions, including LPPC (F(1,17)=39.563, p<0.0001, $\eta_{G}^{2}=0.268$), RPPC (F(1,17)=34.632, p<0.0001, $\eta_{G}^{2}=0.38$), and CZ (F(1,17)=39.945, p<0.0001, $\eta_{G}^{2}=0.378$). Importantly, a significant main effect of cTBS stimulation on amplitude was observed (F(2,34)=5.888, p<0.01, $\eta_{G}^{2}=0.036$), with post hoc analyses indicating a significantly lower amplitude in sham stimulation compared to RPPC stimulation ($p_{\text{adj}}<0.05$, $\eta^{2}=0.352$). However, the absence of a significant effect of Time (F(1,17)=3.822, p=0.067) suggests that pre-test and post-test amplitude values did not differ substantially. Furthermore, the two-way interactions between Level × Stim, Level × Time, and Stim × Time were not significant (F(2,34)=0.081, p=0.922; F(1,17)=0.028, p=0.869; F(2,34)=0.387, p=0.682, respectively). Similarly, the three-way interaction between Level × Stim × Time was non-significant (F(2,34)=0.993, p=0.381). Pairwise comparisons for amplitude across pre-test and post-test conditions revealed no statistically significant differences among LPPC, RPPC, and CZ stimulation conditions. These findings provide further evidence supporting the null hypothesis regarding amplitude modulation.

The tau parameter (τ), reflecting the latency to the peak of automatic activation, displayed an unexpected pattern, see Fig 7. The tau parameter was significantly lower in global trials compared to local trials (F(1,32)=14.567, p<0.0001, $\eta_{G}^{2}=0.039$), which contrasts with the behavioral results. This suggests a possible discrepancy in the timing of activation between global and local processing mechanisms, indicating that the effect of local-to-global interference was higher than global-to-local interference. The effect of cTBS stimulation on tau was not statistically significant (F(2,64)=2.010, p=0.142), nor were the two-way interactions between Level × Stim, Level × Time, and Stim × Time (F(2,64)=2.213, p=0.118; F(1,32)=0.182, p=0.673; F(2,64)=1.752, p=0.182, respectively). However, a significant main effect of Time (F(1,32)=12.181, p<0.001, $\eta_{G}^{2}=0.016$) revealed that tau values were higher in post-test conditions compared to pre-test conditions. This time-dependent effect was specific to RPPC stimulation (F(1,32)=12.459, p=0.001, $\eta_{G}^{2}=0.067$), as no significant changes were observed for LPPC (F(1,32)=2.234, p=0.145) or CZ (F(1,32)=0.054, p=0.817).

The drift rate, which represents the rate of evidence accumulation, showed no significant differences between global and local trials (F(1,21)=0.979, p=0.334) or between pre-test and post-test conditions (F(1,21)=0.246, p=0.625), see Fig 7. Additionally, no significant effects of cTBS stimulation on drift rate were found (F(2,42)=1.009, p=0.373). Although a significant interaction between Level and Stim (F(2,42)=7.610, p=0.002, $\eta_{G}^{2}=0.033$) was detected, post hoc analyses indicated this effect was confined to local conditions. Under local conditions, significant differences were observed between LPPC and CZ stimulation ($p_{\text{adj}}<0.05$, $\eta^{2}=0.382$) and between CZ and RPPC stimulation ($p_{\text{adj}}<0.05$, $\eta^{2}=0.465$). However, the lack of significant Time effects suggests these results may not reliably reflect sustained differences.

The boundary parameter, indicating the evidence threshold required for decision-making, demonstrated a significant main effect of Level (F(1,20)=59.196, p<0.0001, $\eta_{G}^{2}=0.1$), with higher boundaries for local trials (mean = 82.25) compared to global trials (mean = 72.35), see Fig 7. This difference was consistent across all stimulation conditions: LPPC (F(1,20)=16.596, p<0.001, $\eta_{G}^{2}=0.072$), RPPC (F(1,20)=25.61, p<0.0001, $\eta_{G}^{2}=0.135$), and CZ (F(1,20)=13.373, p<0.005, $\eta_{G}^{2}=0.097$). A significant main effect of Time (F(1,20)=12.487, p<0.005, $\eta_{G}^{2}=0.034$) showed that boundaries were higher in pre-test conditions compared to post-test conditions. However, cTBS stimulation did not significantly influence boundary values (F(2,40)=0.887, p=0.42), nor did interactions involving Level, Time, and Stim reach significance.

Finally, the residual time, capturing non-decision processes such as stimulus encoding and motor execution, was significantly longer in local trials compared to global trials (F(1,28)=114.965, p<0.0001, $\eta_{G}^{2}=0.281$) and in pre-test compared to post-test conditions (F(1,28)=32.913, p<0.0001, $\eta_{G}^{2}=0.061$), see Fig 7. However, residual time was not significantly affected by cTBS stimulation (F(2,56)=0.308, p=0.736), nor were any significant interactions involving stimulation, level, or time detected.

Overall, the cognitive modeling results align with behavioral findings, particularly in capturing level-dependent differences in parameters such as amplitude and boundary. However, cTBS stimulation effects were generally inconsistent across parameters and testing sessions, providing limited evidence for its influence on global or local processing mechanisms.

#### Composite face task.

**Fig 8:**
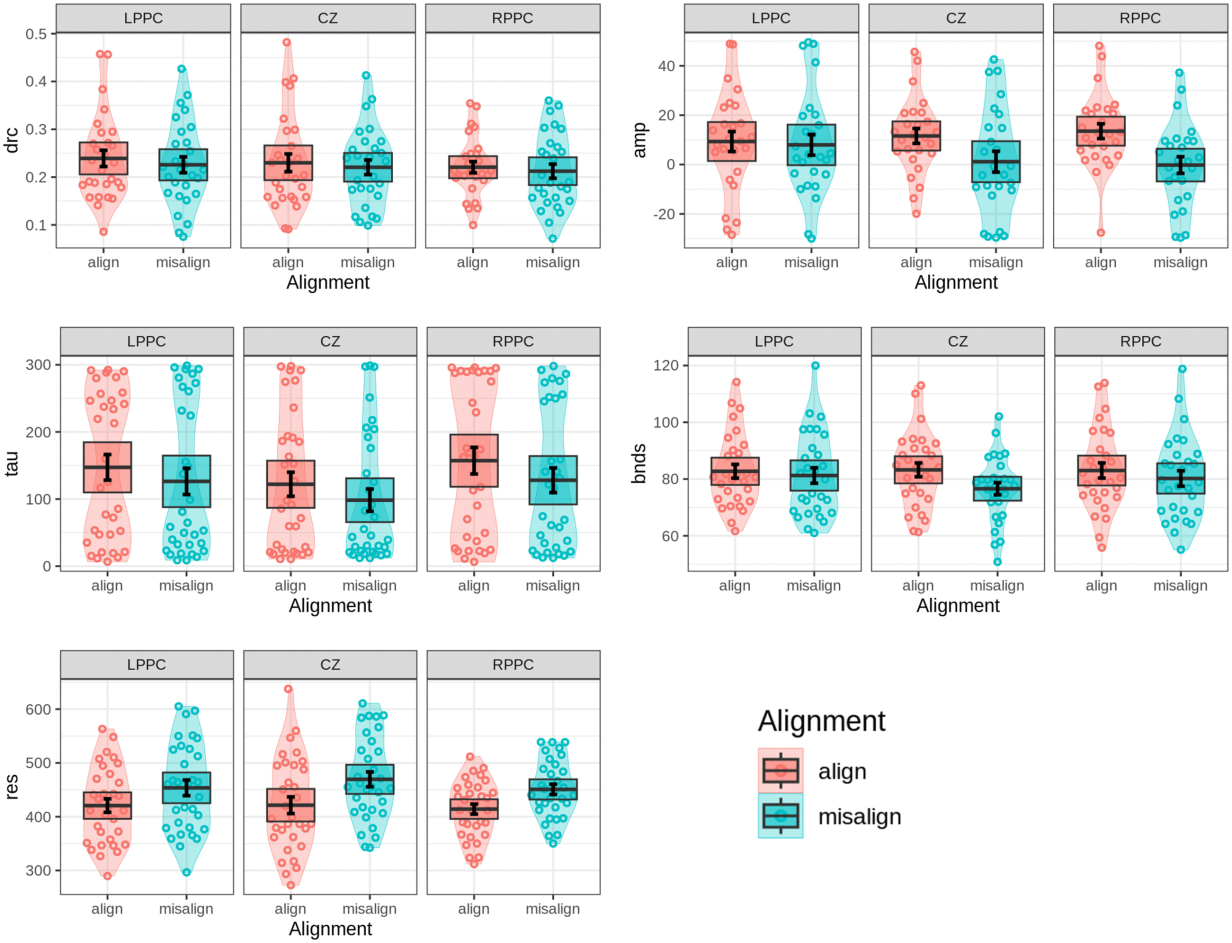
Parameter values of the DMC model for the composite face task. Violin plots illustrate the distributions of key parameters—*drc* (drift rate), *amp* (amplitude), *tau* (latency), *bnds* (decision boundary), and *res* (residual time)—across distinct stimulation (LPPC, RPPC, CZ) and alignment (aligned, misaligned) conditions. Overlaid data points reflect individual participant values, while error bars indicate one standard error around the mean. Shaded boxes represent the 95% confidence intervals assuming a normal distribution for the mean. These visualizations highlight variations in cognitive processing dynamics under different experimental conditions.

To investigate the impact of alignment and cTBS stimulation on cognitive modeling parameters underlying face processing, a two-way repeated-measures ANOVA was conducted on the DMC model parameters with Alignment (aligned, misaligned) and Stim (LPPC, RPPC, CZ) as factors. The analysis of the amplitude parameter, which reflects the maximum effect of automatic activation, revealed a significant main effect of Alignment (F(1,25)=10.676, p<0.005, $\eta_{G}^{2}=0.051$), indicating that amplitude was substantially lower in misaligned trials compared to aligned trials (mean amplitude: 2.971 vs. 11.4756), see Fig 8. This result underscores the critical role of holistic face processing, as alignment significantly modulates automatic activation.

In contrast, cTBS stimulation showed no significant effect on amplitude across stimulation conditions (F(2,50)=0.235, p=0.792), supporting additional evidence in favor of the null hypothesis. Similarly, the Alignment × Stim interaction was non-significant (F(2,50)=1.382, p=0.261), suggesting that alignment effects on amplitude were consistent across all stimulation conditions. To further explore potential differences, a series of paired t-tests were conducted to compare amplitude values across experimental and sham groups. Pairwise mean comparisons between LPPC and CZ, LPPC and RPPC, and RPPC and CZ revealed no statistically significant differences under either alignment condition, providing additional support for the null hypothesis regarding stimulation effects on amplitude, as illustrated in Fig 8.

The tau parameter, representing the latency to the peak of automatic activation, exhibited no significant main effect of Alignment (F(1,32)=2.168, p=0.151) or cTBS stimulation (F(2,64)=2.203, p=0.119). Additionally, the interaction between Alignment × Stim on tau values was not significant (F(2,64)=0.032, p=0.968), indicating that neither alignment nor stimulation meaningfully influenced the timing/temporal dynamics of automatic activation in this task.

Similarly, the drift rate parameter did not differ significantly between aligned and misaligned conditions (F(1,27)=1.622, p=0.214) or across stimulation days (F(2,54)=1.032, p=0.363), as shown in Fig 8. The Alignment × Stim interaction also remained non-significant for drift rates (F(2,54)=0.061, p=0.941). These findings provide additional evidence that cTBS stimulation had no discernible impact on the cognitive processes indexed by drift rate.

The boundary parameter showed a significant main effect of Alignment (F(1,28)=9.870, p<0.005, $\eta_{G}^{2}=0.018$), see Fig 8. Boundary values were higher in aligned trials (mean = 82.984) compared to misaligned trials (mean = 79.3443), emphasizing the influence of alignment on decision thresholds. However, this alignment effect was only significant under sham stimulation conditions (F(1,28)=16.847, p<0.001, $\eta_{G}^{2}=0.07$). cTBS stimulation did not significantly influence boundary estimates (F(2,56)=0.417, p=0.661), nor was the Alignment × Stim interaction significant (F(2,56)=2.312, p=0.108). These results further indicate that the decision thresholds were not affected by experimental manipulation of stimulation conditions.

Consistent with behavioral results, the residual time parameter demonstrated a significant main effect of Alignment (F(1,31)=41.408, p<0.0001, $\eta_{G}^{2}=0.071$). Residual times were longer in misaligned trials compared to aligned trials, reflecting the increased difficulty of processing misaligned faces. This alignment effect was significant across all stimulation conditions: LPPC (F(1,31)=9.211, p<0.01, $\eta_{G}^{2}=0.045$), RPPC (F(1,31)=20.034, p<0.0001, $\eta_{G}^{2}=0.108$), and CZ (F(1,31)=29.819, p<0.0001, $\eta_{G}^{2}=0.08$), as shown in Fig 8. Despite these alignment effects, cTBS stimulation had no significant main effect on residual time (F(2,62)=0.869, p=0.425), nor was there a significant interaction between Alignment × Stim (F(2,62)=0.824, p=0.443).

Taken together, the cognitive modeling results for the composite face task highlight the critical role of alignment in modulating automatic activation, decision thresholds, and non-decision times, thereby reinforcing the importance of holistic processing in face perception. However, the absence of significant effects of cTBS stimulation across all parameters suggests that non-invasive brain stimulation did not alter the underlying cognitive mechanisms of holistic face processing in this study.

## Discussion

Several studies have highlighted that specific experimental manipulations can induce shifts in global and local processing orientations, either enhancing or disrupting them depending on the context and the neural mechanisms involved – including methods such as priming and non-invasive brain stimulation [23,24,79]. Motivated by these findings, this study aimed to investigate whether transcranial magnetic stimulation, specifically using the continuous theta-burst stimulation protocol could causally modulate global and local processing across tasks engaging hierarchical and holistic mechanisms. By targeting the left and right PPC, we sought to explore whether inhibitory stimulation could selectively disrupt the cognitive mechanisms underlying local and global processing, respectively. This work extends prior research by integrating behavioral measures and computational modeling, providing a novel approach to understanding the cognitive and neural mechanisms underlying hierarchical and holistic processing.

Our primary objective was to determine whether inhibitory cTBS could causally disrupt global and local processing. It was hypothesized that stimulation over the right PPC would impair global processing, while stimulation over the left PPC would disrupt local processing. To test these hypotheses, we assessed participants’ performance in two distinct tasks—the hierarchical Navon task and the composite face task—chosen to represent global-local processing and holistic face processing, respectively. We adopted a dual approach that combined behavioral assessments with computational modeling. This approach allowed us to evaluate the extent to which cTBS could influence response accuracy and reaction times while also probing the underlying cognitive mechanisms through the diffusion model of conflict. Importantly, we also investigated whether similar cognitive mechanisms underpin performance in the Navon task and composite face task, providing a broader understanding of hierarchical and holistic processing. While prior studies have explored the neural substrates of global and local processing, to the best of our knowledge, this is the first to integrate cTBS and computational modeling to address these questions comprehensively.

Behaviorally, the findings revealed some nuanced trends. While subtle changes in response accuracy post-stimulation were noted for both the left and right PPC during the Navon task, these differences were not statistically significant when compared to the sham group, suggesting limited effects of cTBS on overall accuracy in either global or local processing. However, response times offered more distinctive insights. Stimulation of the left PPC led to disruptions in local processing under incongruent conditions of the Navon task, evident in longer response times. In contrast, right PPC stimulation did not produce significant changes in response times for either congruent or incongruent conditions, relative to the sham group. These findings underscore the lateralized nature of PPC contributions to processing interference.

Notably, comparisons between left and right PPC stimulation effects revealed meaningful differences in response times during incongruent local blocks of the Navon task. This lateralized effect highlights the specificity of PPC regions in mediating the resolution of processing conflicts. By contrast, in the composite face task, no significant differences in either accuracy or response times were observed following stimulation of either PPC region relative to sham. This lack of effect may reflect the inherent complexity or robustness of holistic face processing mechanisms, which could rely on distinct neural circuits or broader networks that are less susceptible to localized disruption by cTBS.

These findings provide critical insights into the neural mechanisms underlying hierarchical and holistic processing. Patterns in both tasks suggest that shared holistic mechanisms may underpin their performance, aligning with theoretical models that propose common neural and cognitive foundations. The asymmetric effects observed in the Navon task align with prior evidence of hemispheric specialization, where the left PPC plays a more prominent role in processing local details, while the right PPC is associated with global processing. The absence of significant effects on global processing following right PPC stimulation suggests that global processing may involve more distributed or resilient neural networks, which are less susceptible to disruption by cTBS.

The results of the composite face task, as noted, further underscore the complexity of holistic processing mechanisms. The lack of significant effects of cTBS stimulation suggests that the neural substrates of holistic face processing may be robust to external manipulations or require different stimulation parameters to elicit measurable changes. This finding raises important questions about the sensitivity of the composite face task to experimental manipulations and underscores the need for future studies to refine methodological approaches for probing the neural correlates of holistic processing.

The integration of computational modeling provided a deeper understanding of these behavioral patterns. Using the DMC, this study explored the nuanced dynamics of local and global interference underlying decision-making processes in both tasks. Although the behavioral data suggested limited effects of cTBS, the DMC model revealed subtle alterations in decision parameters, such as drift rates and decision thresholds, which indicate shifts in cognitive strategies that might otherwise remain undetected. In particular, the amplitude and boundary parameters showed partial alignment with the behavioral findings, particularly in capturing differences in interference patterns. These findings emphasize the importance of computational approaches in capturing latent effects that extend beyond observable performance metrics.

While these findings are promising, they represent only the initial steps of the analysis. The discussion will now turn to a more detailed examination of the behavioral and computational modeling results, exploring the implications of these findings and situating them within the broader context of hierarchical and holistic processing.

### Effects of cTBS stimulation on global/local dichotomy

The Navon paradigm remains one of the most widely utilized methods to investigate global and local processing orientations [3]. Consistent with previous research, our findings confirmed the expected global precedence effect in mean response times, with participants processing global letters faster than local letters [123, 125, 142, 143]. This effect was more pronounced in the sham group, where global letters were processed more efficiently than local letters, and global information exerted greater interference on local processing than the reverse. Moreover, the congruency effect—characterized by superior performance in congruent trials relative to incongruent ones—was evident, aligning with previous studies [125,143,144]. Importantly, a Level × Congruency interaction was observed, reflecting an asymmetric pattern of interference effects wherein the congruency effect was more pronounced in local trials than in global trials. This asymmetry corroborates earlier research findings and underscores the dominance of global interference in hierarchical stimuli processing [35,123,124,142,145].

To gain deeper insights into the neurocognitive mechanisms underlying these behavioral effects, the diffusion model for conflict was employed to analyze RT distributions. Model parameters revealed that global-to-local interference was most prominently reflected in the amplitude parameter, which was significantly higher in local trials compared to global trials. This supports the notion that global processing disrupts local processing (i.e., global-to-local interference) more effectively than the reverse, consistent with earlier findings [123,125]. The tau parameter, indicative of the latency to the peak of automatic activation, exhibited a lower value in global trials than in local trials. Although this result presented an inconsistency with the behavioral outcomes, it highlights a potential divergence in the timing of cognitive processes governing global and local task performance. Boundary and non-decision time parameters further reinforced the global advantage effect, with higher boundary values and longer non-decision times observed in local trials. These results suggest that participants adopt more conservative decision thresholds and require more time for perceptual encoding and motor execution when identifying local letters. Collectively, these findings reveal how attentional allocation, decision-making thresholds, amplitude, and processing times contribute to the hierarchical organization of visual stimuli.

Given these baseline findings, the study sought to evaluate whether cTBS could modulate global and local processing. Drawing from previous studies [78,79,100,107], it was hypothesized that cTBS over the RPPC would disrupt global processing, whereas cTBS over the LPPC would impair local processing. However, the results diverged from these predictions. Neither post-stimulation accuracy nor RT analyses revealed significant differences between RPPC stimulation and sham conditions. Notably, cTBS over the LPPC resulted in slower responses to local targets under incongruent conditions, partially supporting the alternative hypothesis. Additionally, RT analyses highlighted credible differences between LPPC and RPPC stimulation in local block tasks under incongruent conditions, with LPPC stimulation eliciting longer RTs compared to RPPC stimulation.

This finding supported the alternative hypothesis that cTBS can modulate local and global processing, although the effect was more noticeable when comparing the active group rather than the sham group. Additionally, this outcome suggests that cTBS over the right PPC was insufficiently impactful to demonstrate a significant difference compared to the sham group. The absence of a pronounced effect of RPPC stimulation on global processing may stem from methodological or neuroanatomical factors, including the distributed and bilateral nature of global processing networks. Such networks may be less susceptible to disruption by localized cortical stimulation.

A critical consideration is the potential methodological discrepancies between this study and earlier investigations. Variations in task design, stimulus properties, and stimulation protocols likely contribute to the observed inconsistencies. We employed the conventional Navon hierarchical letters paradigm [3] as used by [78], to investigate the effect of stimulation purely on global and local processing. However, [107] utilized compound letters with a divided attention task, wherein each stimulus contained a target letter at one level and a distractor at another. They evaluated global/local switching by regularly altering the target letter level, which is distinct from the task design used in our study. Moreover, [79,100] used a modified version of Navon letters, incorporating blurred and non-blurred displays to manipulate the saliency of local and global levels. In these studies, global saliency was enhanced in blurred displays, whereas local saliency was elevated in non-blurred displays. Similarly, [146] introduced a different method by scrambling hierarchically organized visual stimuli, manipulating global Gestalt perception through varying degrees of element displacement at the local level, ranging from 20% to 80%. This experimental design contrasts with our conventional Navon task, highlighting a potential source of variation in outcomes.

Moreover, differences in the duration of stimulus presentation further complicate direct comparisons across studies. Previous research has shown that changes in stimulus characteristics, such as exposure duration, and the number and size of elements in hierarchical figures, significantly affect the presence of the global advantage [7,30,34].

Beyond task design and stimuli, the stimulation protocols themselves might contribute to the disparate results. In this study, cTBS was delivered using the Super Magstim Rapid2 stimulator with a 70-mm figure-of-eight coil, a setup consistent with earlier studies [78,79,100,107,146]. However, key differences in stimulation parameters may explain the discrepancies. For instance, [78] applied low-frequency TMS over the left and right PPC at 1 Hz in both right- and left-handed participants at an intensity equal to 90% of their motor threshold. Their findings, contrary to the current study’s hypotheses, did not reveal significant RT differences between stimulation days and pre-stimulation conditions, suggesting that the chosen protocol did not robustly affect both local and global processing. An increase in global-to-local interference was only observed following rTMS over the left PPC, but no corresponding local-to-global interference effects emerged from right PPC stimulation. These results suggest that 1 Hz rTMS with 600 pulses per train had a limited impact on local and global processing in right-handed individuals, providing insights into the PPC’s functional specialization.

In another study, [107] attempted to replicate the findings of [78]. Their approach involved applying 1 Hz rTMS to the left and right PPC (with CZ as a control site) and measuring response times to global and local targets. Consistent with earlier findings, rTMS over the left PPC enhanced global-to-local interference and accelerated responses to global stimuli compared to the control condition. However, response times to local stimuli were not modulated by right PPC stimulation, nor did the researchers find evidence for target level switching effects. These results further highlight the limitations of 1 Hz rTMS as an inhibitory protocol for modulating local and global processing.

In contrast, [79,100] adopted rhythmic TMS protocols, delivering short bursts of five pulses at theta, beta, or alpha band frequencies. Their results revealed that alpha band TMS over the right PPC impaired global processing, whereas similar stimulation over the left PPC disrupted local processing. The rhythmic nature of this protocol allowed for precise temporal alignment with neural oscillations, but its short-lasting effects limited its utility for the goals of the present study. The online stimulation protocol used in their research necessitated application immediately before stimulus presentation, making it unsuitable for the longer-lasting effects desired in this investigation. To address this limitation, cTBS600 was employed here, delivering 600 pulses over 40 seconds to produce inhibitory effects lasting up to 60 minutes. This protocol has been shown to suppress motor-evoked potentials for an extended duration, as demonstrated by [147], who found that 300-pulse cTBS increased excitability, whereas 600-pulse cTBS induced inhibition. Therefore, depending on the number of pulses employed during the stimulation, cTBS stimulation may be able to produce either excitatory or inhibitory after-effects [147,148].

Another study by [146] explored whether applying TMS over the temporoparietal junction (TPJ) could modulate global Gestalt perception. Using cTBS300 at 80% of the active motor threshold, they observed that bilateral TPJ stimulation unexpectedly enhanced accuracy and reduced response times, despite prior indications that cTBS over the motor cortex could inhibit such effects [109,149]. Their findings raise important questions about the dose-dependent effects of cTBS protocols and the influence of stimulated cortical regions on behavioral outcomes. While cTBS300 induced suppression for approximately 20 minutes [109], cTBS600 was selected for this study to achieve longer-lasting effects suitable for dual-task experiments. However, the results suggest that the behavioral effects of cTBS may vary depending on the cortical area targeted and the number of pulses employed.

In addition to stimulation protocol, differences in the stimulation site may account for the observed discrepancies. Global and local processing has been widely regarded as lateralized within the brain [150], with the right PPC implicated in global processing and the left PPC associated with local processing. However, there is ongoing debate in the literature regarding the precise hemispheric lateralization of these processes [9, 151]. Numerous studies employing brain imaging, neuroimaging, EEG, or patient data have provided evidence supporting this hemispheric specialization. These studies suggest that global processing predominantly involves the right parietal cortex, while local processing is largely driven by the left parietal cortex [78,79,87,88,90,92,100,150].

For instance, an fMRI study by [101] found that activity in the anterior intraparietal sulcus (aIPS) was strongly correlated with the conscious perception of a hierarchical stimulus, whether as a whole or as individual elements. When an inhibitory TMS protocol was applied to disrupt activity in this region, the perceptual duration of global Gestalt features was substantially reduced without impairing the perception of local elements. These findings underscore the critical role of the right posterior parietal cortex, including aIPS, in attentional allocation to global stimulus aspects [78,79,99,100].

Interestingly, several studies have demonstrated that this hemispheric lateralization can be manipulated or even reversed depending on specific experimental parameters [152]. Factors such as stimulus category [9], spatial frequency [153], and stimulation frequency [79] have been shown to influence hemispheric dominance. Additionally, research suggests that the TPJ is bilaterally involved in processing the global Gestalt of visually organized inputs, and bilateral lesions in this region can result in simultanagnosia [146,154]. These findings highlight the complexity of neural mechanisms underlying global and local processing, suggesting that lateralization is not fixed but rather context-dependent.

Consistent with prior research [78,79,100], we applied unilateral TMS stimulation to the left and right posterior parietal cortex to investigate its impact on local and global processing. These regions have been implicated in both non-spatial and spatial aspects of visual attention [155–157]. By stimulating the P3 and P4 regions defined by the 10/20 EEG positioning system, this study aimed to provide insights into the functional specialization of the PPC. Although the approach differed from neuronavigation-based studies that used individual structural MRI scans for precise spatial targeting [79,100,158], the 10/20 system provided a practical and replicable alternative [78,107]. It is worth noting, however, that the spatial resolution of TMS using a figure-of-eight coil is approximately 4.5 cm in diameter, potentially limiting the precision of cortical stimulation [159,160]. Nevertheless, neuronavigation systems can enhance reliability and reduce variability across participants [158]. Despite these limitations, both targeting methods may yield comparable results due to the relatively broad area influenced by TMS stimulation.

The variability in findings across studies may also be attributed to differences in experimental design. In contrast to earlier investigations that employed between-subjects designs with smaller sample sizes [79], this study used a within-subjects design involving a larger sample. This methodological choice enhances statistical power by reducing inter-individual variability. Within-subjects designs are particularly advantageous for detecting subtle effects, as each participant serves as their own control, thereby eliminating confounding variables associated with between-group differences [161]. Consequently, the current study provides a robust framework for examining the effects of cTBS stimulation on local and global processing.

In summary, differences in stimulation protocol, site, and experimental design likely underlie the discrepancies between the current findings and previous research. While cTBS over the LPPC selectively modulated local processing, as evidenced by prolonged RTs under incongruent conditions, the effects of cTBS on global processing were less pronounced. Notably, significant differences in post-stimulation response times were observed between the left and right posterior parietal cortex in local processing, underscoring the lateralized contributions of these regions. However, the overall inconsistency with prior studies suggests that the impact of cTBS stimulation is context-dependent and may be influenced by methodological variations. To further probe these effects, DMC model analyses were employed to dissect RT distributions and accuracy rates, revealing several critical findings. First, cTBS stimulation produced significant variations in amplitude parameters, with lower values observed in sham stimulation compared to RPPC stimulation. Yet, the lack of time-dependent differences across pre-test and post-test conditions raises questions about the reliability of this result. Second, the interaction between Level and Stimulation in drift rate parameters pointed to differential effects across stimulation sites under local conditions. However, the absence of significant temporal changes in drift rates casts doubt on the robustness of these findings. Additionally, time emerged as a significant factor influencing multiple parameters. Tau values were elevated in post-test conditions, while boundary separation and non-decision time were higher in pre-test conditions. These patterns align with behavioral evidence, suggesting the influence of fatigue or practice effects, particularly in conflicting conditions. Despite these insights, the DMC model analyses failed to uncover a consistent main effect of cTBS stimulation on global and local processing, underscoring the complexity of these neural dynamics and highlighting the importance of protocol standardization and task designs for future investigations of the complex interactions between brain stimulation and cognitive processing pathways.

### Effects of cTBS stimulation on holistic face processing

While most studies investigating local and global processing have focused on hierarchical Navon letters, the current research expanded this scope by employing the composite face effect. The composite face task provides compelling evidence for holistic face processing, wherein the global form of a face is prioritized over its local features [56,70]. Previous research has highlighted similarities between the cognitive mechanisms underlying composite face processing and Navon hierarchical letters. Both paradigms are thought to engage holistic processes where the global structure, such as the overall face configuration, takes precedence over local features like individual facial elements [35–37]. By incorporating the composite face task, this study aimed to investigate whether these tasks share underlying neurocognitive mechanisms and to evaluate whether inhibitory cTBS protocols would exert comparable effects on both.

Central to the composite face task is the interaction between alignment and congruency, which serves as an indicator of holistic processing. Consistent with prior research [45, 65, 69, 70], this study replicated the congruency effect, with faster and more accurate responses in congruent trials compared to incongruent ones. Additionally, the alignment × congruency interaction demonstrated that the congruency effect was diminished when faces were misaligned, indicating that the bottom half interfered with judgments of the top half only in aligned conditions. This pattern underscores the holistic nature of face processing, aligning with earlier findings [45,70–72].

Fitting the DMC model to RT distributions provided additional insights into the cognitive mechanisms underpinning these results. Specifically, three key parameters were implicated in the composite face effect. First, the amplitude parameter was significantly higher in aligned trials than in misaligned trials, mirroring behavioral findings and suggesting that irrelevant information from the bottom half of composite faces interfered more in aligned conditions, which is supported by findings from previous studies [24,45,70–72]. Second, boundary separation was greater in aligned conditions, reflecting more conservative decision-making when the meaningful configuration of a face was intact [47,56,63,66], paralleling findings in the Navon task where higher boundaries were observed in local trials. This implies that disrupting the face’s meaningful configuration hampers holistic processing [47,56,63,66]. Lastly, non-decision time analyses showed longer durations in misaligned trials, reflecting slower responses in these conditions, consistent with the behavioral data. Together, these findings suggest that the composite face effect is driven by conflict processing (amplitude), decisional mechanisms (boundary), and perceptual encoding and motor responses (non-decision time). Notably, these mechanisms parallel those identified in the Navon task, further supporting the hypothesis that both tasks engage similar cognitive processes.

The study hypothesized that cTBS over the left PPC would impair the recognition of individual face components, whereas cTBS over the right PPC would disrupt holistic face processing. Contrary to expectations, no significant differences in mean accuracy or RT were found between the sham group and either cTBS stimulation group for aligned trials. However, mean accuracy analyses revealed that cTBS over the left PPC yielded marginally more accurate responses in congruent misaligned trials compared to the CZ and right PPC stimulation groups. While these results suggest a potential selective effect of LPPC stimulation, they were insufficient to confirm the alternative hypothesis, particularly given that theoretical predictions emphasized stronger effects on aligned trials. Moreover, DMC model analyses provided robust evidence in favor of the null hypothesis, revealing no significant effect of cTBS on model parameter estimates.

To the best of our knowledge, this study is the first to evaluate the effects of cTBS, as an inhibitory protocol, on holistic face processing as measured by the composite face task. Previous research investigating manipulations of the composite face effect has relied on two primary methods: priming with Navon stimuli to bias local or global processing [23–25,75] and transcranial direct current stimulation (tDCS) [80,81]. However, both approaches have yielded inconsistent findings. For instance, some research found that local priming improved part-based face recognition, while others showed the opposite effect, with global priming enhancing performance [23,24]. Methodological differences, such as whether accuracy or RT was prioritized, likely account for these discrepancies. Additionally, the transient nature of priming effects [77,114] led to its exclusion from this study, which sought a longer-lasting modulation of neural networks.

Non-invasive brain stimulation, such as tDCS, offers another method to manipulate the composite face effect. However, existing studies using tDCS have yielded contradictory outcomes. For instance, [80] observed reduced composite face effects with tDCS over the occipito-temporal cortex, while others found no significant impact on holistic processing [81,162]. Differences in stimulation sites, protocols, and task designs may explain these inconsistencies. For example, [80] applied online tDCS over the occipito-temporal cortex during an untimed composite face task, emphasizing accuracy over RT. In contrast, [162] reported no significant effects of anodal tDCS over the occipital face area in a timed task. These results suggest that tDCS effects on cognitive tasks may be modest and context-dependent [163]. Unlike Yang et al.’s online stimulation approach, our use of the inhibitory cTBS protocol aimed for a prolonged aftereffect, potentially offering a more sustained alteration of neural activity. However, the timing of task administration post-stimulation may have influenced our findings, as peak cTBS effects could occur later than the composite face task presentation.

A similar single-blind, within-subjects design was employed in another study by [162, Experiment 1], where the composite face task was presented both before (pre) and after (post) real and sham anodal tDCS stimulation over the occipital face area (OFA), a key region within the occipito-temporal cortex. Unlike the findings of [80], real anodal tDCS had no effect on holistic face processing as measured by the composite face task, a result consistent with the findings of the current study. Additionally, a more recent between-subjects study found that the composite face effect was not significantly affected by online anodal tDCS delivered for 10 minutes over the left dorsolateral prefrontal cortex (cathodal: right eyebrow, anodal: Fp3) [81]. These findings suggest that achieving significant cognitive changes through a single tDCS session may be challenging. Meta-analyses of tDCS effects on cognitive functions further support this, indicating small-to-medium effect sizes [163].

Recognizing these limitations, the current study adopted TMS as an alternative approach to investigate the modulation of holistic face processing, aiming for a longer-lasting inhibitory effect on specific neural circuits. Unlike tDCS, which has been extensively studied but remains inconsistent in its outcomes, the effects of cTBS stimulation specifically delivered at P3 and P4 sites on the composite face effect have not been previously explored. The posterior parietal regions were targeted in this study to modulate attentional processes via an inhibitory protocol, aiming to disrupt both global and local attention in tasks like Navon letters and composite faces. Interestingly, this study—similar to many previous tDCS investigations—found that manipulating the composite face effect using an alternative brain stimulation method did not yield a statistically significant difference, suggesting that the cTBS protocol used might not have been optimal for this purpose.

The null findings in this study may be partially explained by the characteristics of the stimulation protocol. It is possible that the stimulation effect required more time to fully manifest than the experimental design allowed. According to [109], reductions in motor evoked potential (MEP) amplitudes following cTBS grow progressively over time. MEPs remain relatively unchanged during the initial minutes post-cTBS but decline steadily, peaking at approximately 25 minutes (for 20-second cTBS) to 60 minutes (for 40-second cTBS), depending on the cTBS protocol duration. Recent work by [164], investigating the effects of cTBS on neuronal activity in the parietal cortex of rhesus monkeys, demonstrated a similar progressive reduction in neuronal excitability, with maximum effects observed 30–50 minutes post-stimulation. These findings closely align with the human MEP amplitude changes reported by [109]. Such delayed effects appear to correlate with an increase in GABA, an inhibitory neurotransmitter, as demonstrated in the human motor cortex using magnetic resonance spectroscopy [165], suggests that the full effects of cTBS might not have been captured within our immediate post-stimulation testing window.

In conclusion, the combined findings of this study and prior research suggest that single-session brain stimulation techniques, such as cTBS and tDCS, may not produce substantial changes in the perceptual processing of faces as measured by the composite face task. Future studies should explore optimized stimulation protocols, including adjustments to timing and task design, to better evaluate the potential for brain stimulation to influence holistic face processing.

### Similarity between Navon letters and composite faces

Navon hierarchical letters and composite faces have long been used as paradigms for investigating holistic processing in visual cognition. The Navon task emphasizes the precedence of global forms over local elements in hierarchical stimuli, while the composite face task investigates the integration of facial features into a unified percept. Numerous studies have proposed that these two paradigms rely on the same holistic processing mechanisms, with deficits in one domain often extending to the other [35,36]. For instance, the global precedence effect in Navon letters parallels the holistic processing advantage in composite faces, wherein global configurations dominate over local features. However, contradictory findings have challenged this assumption, with some evidence suggesting distinct underlying mechanisms for Navon letters and composite faces [74,124]. A recent study by [145] aimed to directly evaluate this relationship using parameters of the Linear Ballistic Accumulator (LBA) model [166]. Their findings suggested that Navon letters and composite faces are governed by separate psychological processes. This conclusion challenges the assumption of shared holistic mechanisms, prompting further investigation into the nature of these cognitive processes.

To further investigate whether Navon letters and composite faces stem from similar holistic mechanisms, our study applied cTBS stimulation as a manipulation tool to investigate the neural and cognitive underpinnings of this relationship. The behavioral and cognitive modeling outcomes from the Navon task indicated that cTBS over the left or right PPC did not produce strong effects compared to the sham condition. Similarly, the composite face task showed no significant influence of cTBS on holistic face processing. If these tasks indeed share holistic processes, then both should exhibit comparable susceptibility to manipulative factors, such as inhibitory brain stimulation. Our findings, supported by analyses of parameters derived from the DMC, provided partial support for this hypothesis, indicating potential overlaps in the cognitive mechanisms underlying both tasks particularly when holistic processing is disrupted.

The DMC model analyses revealed consistent patterns across both tasks, particularly in the amplitude parameter, which quantifies the influence of irrelevant information. In the Navon task, amplitude values were significantly higher in local trials than in global trials, reflecting stronger interference from global elements during local processing. Similarly, in the composite face task, aligned trials yielded higher amplitude values than misaligned trials, indicating greater interference from irrelevant facial components when faces were perceived as unified wholes. These findings align with previous studies on global-to-local interference and holistic face processing [37,70], reinforcing the idea that both tasks engage similar cognitive processes when dealing with conflicting conditions.

The boundary parameter provided additional insights into shared mechanisms. In the Navon task, participants demonstrated higher boundary values in local trials than in global trials, reflecting a more conservative decision-making strategy during global-to-local interference. This pattern was mirrored in the composite face task, where aligned trials were associated with higher boundary values, suggesting a similar cautious decision-making process under holistic interference. These consistent results indicate that both tasks involve comparable decisional strategies influenced by global configurations.

The non-decision time parameter provided additional support for shared mechanisms. In the Navon task, non-decision times were longer in local trials than in global trials, supporting the precedence of global forms in early visual processing. This pattern was echoed in the composite face task, where misaligned trials had longer non-decision times than aligned trials. Together, these findings highlight the temporal advantage of global configurations in both paradigms, further suggesting shared mechanisms for holistic processing.

Discrepancies between our findings and those of [145] may arise from variations in study design and analytical approaches. While [145] used a between-subjects design to minimize carryover effects, our within-subjects design increased statistical power by reducing inter-individual variability. Additionally, their reliance on the LBA model may have limited their ability to dissociate the nuanced dynamics of conflict processing. The DMC model, employed in this study, offers a more detailed framework by separately modeling task-relevant and irrelevant processes, offering insights into conflict processing that simpler models might overlook.

Delta plots, which track the evolution of congruency effects over time, provided further evidence for the shared mechanisms underlying these tasks. Both Navon letters and composite faces exhibited negative delta plot slopes, indicating that interference effects diminished with longer response times. This finding aligns with the global-to-local hypothesis [167], which posits that global information dominates early stages of processing, while local information becomes more salient with increasing response times. This temporal dynamic was reflected in the decreasing interference observed in both tasks, indicating shared cognitive dynamics. These findings contrast with those of [168], who reported positive delta plot slopes for the composite face task, arguing that it shares processes with paradigms like the Stroop and Flanker tasks. The discrepancy may stem from differences in experimental design or modeling frameworks, underscoring the importance of methodological considerations when interpreting cognitive mechanisms.

Overall, our findings suggest that Navon letters and composite faces engage overlapping cognitive mechanisms, as evidenced by consistent patterns in behavioral and DMC parameter analyses. Both tasks demonstrated similar interference effects, decision-making dynamics, and temporal processing structures, supporting the hypothesis that they are governed by shared holistic processes. While differences in experimental design and modeling approaches may account for conflicting results in the literature, our study provides a robust framework for exploring the relationship between these paradigms. Future research should build on these findings by employing diverse methodologies to further elucidate the cognitive and neural mechanisms underlying global-local processing.

### Limitations and future directions

While the findings of this study underscore the minimal effects of cTBS stimulation on local and global processing in both the hierarchical Navon task and composite face task, several methodological limitations must be acknowledged. These limitations, particularly concerning the stimulation parameters, timing of task presentation, and targeting accuracy, provide critical avenues for future research to refine experimental designs and optimize the effectiveness of cTBS in hierarchical visual processing tasks, thereby advancing our understanding of the underlying cognitive mechanisms.

The method employed to determine the motor threshold (MT) is a significant factor influencing the outcomes of this study. Specifically, the visible twitch (VT) method was used to establish the MT due to its simplicity and feasibility in clinical and experimental settings. However, accumulating evidence indicates that the VT method often overestimates MT compared to the electromyography (EMG)-based approach. This overestimation results in higher stimulation intensities, which may compromise the focality and accuracy of the TMS effects [169–171]. Overstimulation can inadvertently activate non-target cortical regions, leading to unintended effects that dilute the precision and interpretability of experimental outcomes. For instance, [172] demonstrated that the VT method consistently produces higher MT values than the EMG approach. While both methods are reliable across repeated measures, the EMG-based approach offers enhanced precision and reduced off-target stimulation. Using the VT method may have contributed to variability in the outcomes of this study, particularly in tasks requiring precise neural modulation. Future studies should prioritize EMG-based MT determination to enhance the specificity and reliability of TMS-induced effects, ensuring greater accuracy in targeting the desired cortical regions.

The temporal alignment of task presentation with cTBS stimulation is another critical methodological limitation. In this study, the Navon hierarchical and composite face tasks were presented immediately following cTBS stimulation. However, prior research suggests that cTBS effects may not manifest fully until 30–50 minutes post-stimulation, as the inhibitory effects of cTBS on neuronal excitability develop gradually over time [109,164]. Administering tasks immediately post-stimulation may therefore fail to capture the peak effects of cTBS-induced neuronal modulation. For example, [109] observed a progressive reduction in motor-evoked potential amplitudes following cTBS over the motor cortex, with the effects peaking well after the initial stimulation. Similarly, [164] found a comparable time-dependent reduction in neuronal excitability in the parietal cortex of rhesus monkeys. These findings suggest that task administration shortly after cTBS may not align with the window of maximal inhibition. Future studies should delay task presentation by at least 10–30 minutes post-stimulation, allowing sufficient time for the cTBS effects to fully manifest. This adjustment would provide a more accurate assessment of the modulatory effects of cTBS on local and global processing.

Another limitation lies in the coil positioning method. This study employed the 10/20 EEG positioning system, which, while widely used, lacks the spatial precision of neuronavigation-based targeting. Neuronavigation allows for the precise placement of the TMS coil based on individual MRI scans, ensuring accurate stimulation of the intended cortical regions. This approach has been shown to improve measurement reliability, spatial accuracy, and the reproducibility of TMS effects [158,160]. The absence of neuronavigation in this study may have introduced variability in targeting the PPC. Given the relatively broad spatial resolution of TMS with a figure-of-eight coil (approximately 4.5 cm in diameter) [159], even minor deviations in coil positioning could lead to unintended stimulation of adjacent cortical areas. Incorporating neuronavigation in future studies would mitigate these issues, enabling more precise and reliable assessment of cTBS effects on hierarchical visual processing.

Beyond stimulation parameters, task timing, and targeting accuracy, other methodological factors warrant consideration. This study employed a within-subjects design, which enhances statistical power by reducing inter-individual variability. However, expanding the sample size and exploring the effects of repeated stimulation sessions over multiple days could yield more robust and generalizable insights. Repeated sessions may reveal cumulative and long-lasting effects of cTBS that are not apparent in single-session designs.

Additionally, future research should explore alternative task designs and stimulation protocols to optimize the detection of cTBS effects. For instance, combining cTBS with other neuroimaging techniques, such as fMRI or EEG, could provide complementary insights into the neural mechanisms underlying local and global processing.

## Conclusion

The present study aimed to explore the influence of continuous theta-burst stimulation over the left and right posterior parietal cortex on global and local processing in two tasks: the hierarchical Navon and composite face tasks. Despite the anticipated effects of cTBS stimulation in modulating participants’ reliance on global versus local processing, the findings provided no compelling evidence that a single session of cTBS significantly impacts these mechanisms. Both behavioral and cognitive modeling results indicated that Navon hierarchical letters and composite faces share similarities in their underlying holistic processing mechanisms, as evidenced by consistent patterns across DMC parameters. However, the lack of significant modulation by cTBS emphasizes the complexity of these neural mechanisms and suggests limitations in the experimental design, including the timing of task administration post-stimulation and the targeting precision of stimulation sites.

Future investigations should address these limitations by incorporating neuronavigation technologies to enhance the accuracy of TMS coil placement, employing electromyographic methods for motor threshold determination, and optimizing the timing between cTBS application and task performance to capture the full spectrum of stimulation effects. Publishing both positive and negative results from studies exploring diverse stimulation protocols will advance our understanding of cTBS as a tool for cognitive neuroscience and its potential to elucidate the neural dynamics of global and local processing. This work contributes to a growing body of evidence on the challenges of non-invasive brain stimulation in cognitive tasks and highlights the need for refined methodologies to unravel the complexities of hierarchical and holistic visual processing.

## Supporting information

S1 Checklist Inclusivity in global research. (PDF)
